# Global Sensitivity Analysis in Life-Cycle Assessment of Early-Stage Technology using Detailed Process Simulation: Application to Dialkylimidazolium Ionic Liquid Production

**DOI:** 10.1021/acssuschemeng.3c00547

**Published:** 2023-04-21

**Authors:** Husain A. Baaqel, Andrea Bernardi, Jason P. Hallett, Gonzalo Guillén-Gosálbez, Benoît Chachuat

**Affiliations:** † Department of Chemical Engineering, Imperial College London, South Kensington Campus, London SW7 2AZ, United Kingdom; ‡ Sargent Centre for Process Systems Engineering, Imperial College London, South Kensington Campus, London SW7 2AZ, United Kingdom; ¶ Institute for Chemical and Bioengineering, Swiss Federal Institute of Technology, Vladimir-Prelog-Weg 1, Zurich 8093, Switzerland

**Keywords:** uncertainty quantification, global sensitivity analysis, life-cycle assessment, environmental sustainability, ionic liquid production

## Abstract

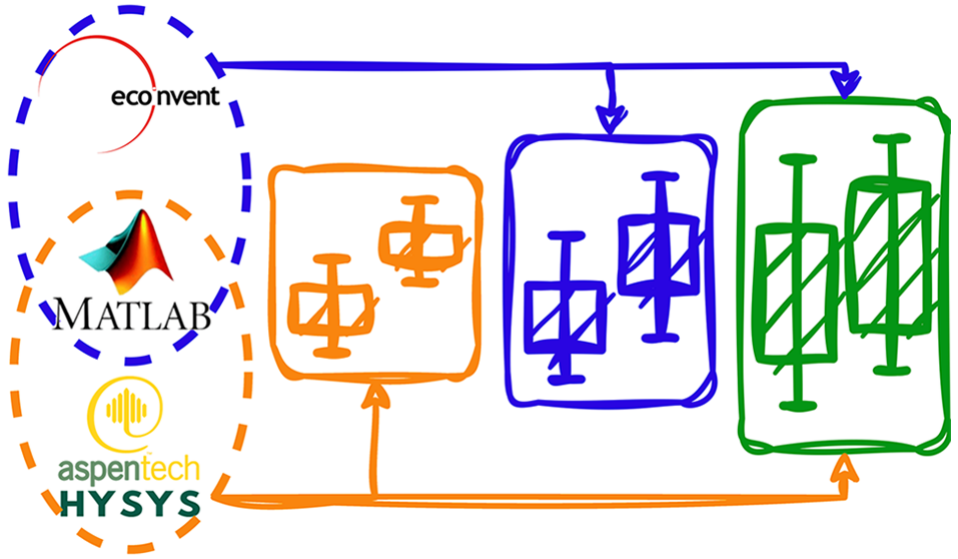

The ability to assess the environmental performance of early-stage technologies at production scale is critical for sustainable process development. This paper presents a systematic methodology for uncertainty quantification in life-cycle assessment (LCA) of such technologies using global sensitivity analysis (GSA) coupled with a detailed process simulator and LCA database. This methodology accounts for uncertainty in both the background and foreground life-cycle inventories, and is enabled by lumping multiple background flows, either downstream or upstream of the foreground processes, in order to reduce the number of factors in the sensitivity analysis. A case study comparing the life-cycle impacts of two dialkylimidazolium ionic liquids is conducted to illustrate the methodology. Failure to account for the foreground process uncertainty alongside the background uncertainty is shown to underestimate the predicted variance of the end-point environmental impacts by a factor of two. Variance-based GSA furthermore reveals that only few foreground and background uncertain parameters contribute significantly to the total variance in the end-point environmental impacts. As well as emphasizing the need to account for foreground uncertainties in LCA of early-stage technologies, these results illustrate how GSA can empower more reliable decision-making in LCA.

## Abstract 2

Combining foreground and background uncertainties in life-cycle assessment of emerging technologies.

## Introduction

The life-cycle assessment (LCA) methodology enables the environmental impact assessment of products and processes throughout their entire life cycle,^1^ covering resource extraction (cradle), production, use, and disposal (grave). LCA follows the ISO 14040 standards and is a prominent environmental assessment method nowadays. It has been applied extensively to support decision-making in both public and private organizations through identifying major hotspots and improvement opportunities. A key strength of LCA lies in the translation of environmental impacts into high-level damage areas, such as human health and ecosystem quality, facilitating the interpretation and communication of the results to stakeholders and decision-makers.

The life-cycle inventory stage of LCA entails the collection of data on mass and energy flows from raw material extraction to process emissions and wastes. For existing processes, such inventory data may be collected directly on-site or retrieved from environmental databases, such as ecoinvent.^2^ In many cases, however, inventory data may be lacking due to low technology readiness of processes or be inaccessible because of confidentiality.^3−5^ These inventory gaps can impede the environmental assessment of chemicals in early development stages, including ionic liquids.^6,7^

Various approaches have been proposed to bridge the gap in inventory data. Streamlined LCA methods aim to predict the life-cycle impacts of a product from readily available information. Calvo-Serrano et al.^8^ developed a streamlined method that relies on linear regression for predicting the life-cycle impacts of chemicals based on their molecular structure, and later refined their approach by including thermodynamic properties and information on the sigma-profile of the molecule.^9^ These regression models were shown to provide accurate predictions for a range of chemicals, including petrochemicals and their derivatives. However, they can lead to large errors with other chemicals and may fail to accurately predict certain life-cycle impacts too, especially impacts that are not directly linked to the descriptors used in the regression models.

Short-cut methods based on stoichiometric yields or simplified models^10,11^ provide an alternative to these simple regression models. Precursor work by Kralisch et al.^12^ led to a simplified LCA method combining lab-scale experiment data with proxy data of similar chemicals as necessary. More recently, Cuéllar-Franca et al.^10^ developed an approach for constructing the life-cycle synthesis tree of a chemical by going all the way back to where data for the most basic precursors are available, or using stoichiometric and basic thermodynamic calculations where data are unavailable. Although convenient to quickly obtain a preliminary estimate, the previous methods may not be suitable for a detailed assessment, especially when comparing products and processes with similar performance indicators. Part of the reason for this is that they omit key process parameters such as heating and cooling duties, process waste, and emissions and process efficiencies.

A more reliable environmental assessment can be supported by detailed process models in order to predict the performance at scale of processes at a low technology-readiness level (TRL), for which industrial process data are yet unavailable.^13^ Commercial process simulators such as Aspen-HYSYS encompass a wide range of unit operations, provide access to accurate thermodynamic property packages, and facilitate mass and heat integration in order to model real-life processes. However, these process models can themselves be subject to large uncertainty.^14−16^ It is of paramount importance, therefore, to quantify these uncertainties and propagate them to the predicted inventories and ultimately the predicted environmental impacts.

The ISO 14044 standard stipulates that a sensitivity analysis should be conducted as part of the LCA framework to identify the most important sources of uncertainty but does not recommend a specific technique. A large body of research has thus been devoted to characterizing, propagating, and analyzing various sources of uncertainty in LCA, using a range of techniques, over the past few decades.^17−20^ Despite this, many LCA studies that build on detailed process simulation simply omit the effect of inventory uncertainties; while many others solely consider uncertainty in the background inventory data,^21−23^ often formulating probability distributions for the inventories using data quality indicators such as a pedigree matrix.^24,25^ A more thorough uncertainty analysis calls for including foreground inventory uncertainties, such as process operating conditions and thermophysical properties, alongside the background inventory uncertainties.^26^ This is especially relevant in comparing processes with similar performance indicators where the corresponding uncertainty ranges might overlap significantly. Moreover, sensitivity analysis could help better understand the effect of key model or process parameters on the predicted foreground inventories, ultimately guiding future experimental work to help reduce this uncertainty.

A popular approach to sensitivity analysis in techno-economic and environmental assessment is one-at-a-time sensitivity analysis, which varies the values of the uncertain input parameters one at a time while keeping the remaining parameters constant at a given reference point and results in a ranking of the uncertain parameters.^27^ This approach works well for models that are mostly separable in their inputs, but the ranking results could be misleading when the level of interactions between the input parameters is more pronounced, a problem that is exacerbated by larger input domains. These limitations can be overcome by applying global sensitivity analysis (GSA),^28^ which accounts for output variations over the entire input domain and has the ability to capture interactions between two or more input parameters. GSA methods do not merely rank the uncertain parameters, but also quantify how much each input parameter contributes to the overall output variance.

Nevertheless, the application of GSA as part of LCA has remained scarce to date.^19,25^ Cucurachi et al.^29^ proposed a protocol for conducting GSA in LCA with a focus on the life-cycle impact assessment (LCIA) stage and in particular on uncertainties in the characterization factors and weighting methods. By contrast, Groen et al.^19^ focused on the life-cycle inventory stage and compared various GSA methods in terms of their effectiveness. A number of recent applications of GSA in LCA include biodiesel production,^30^ building design,^31^ geothermal heating networks,^32^ and advanced photovoltaic cells.^33^ A key challenge in these GSA applications remains the very large number of uncertain input factors, especially when dealing with inventory data.^34−36^ This may require a huge number of samples to compute reliable sensitivity indices and result in high computational burden or even become intractable when a detailed process simulator is used to fill in inventory data gaps, for instance in early-stage technological assessments. It could also explain why the particular combination between GSA and detailed process simulators has not yet been investigated in the LCA literature.

The main objective of this paper, therefore, is to investigate the combination between GSA techniques, LCA databases, and detailed process simulators in the environmental assessment of low TRL technologies. The focus is on analyzing the combined effect of background and foreground inventory uncertainties. A new methodology is introduced, whereby the uncertain background inventories flows, either downstream or upstream of the foreground processes, are lumped in order to reduce the number of factors in the sensitivity analysis and improve computational tractability. A practical implementation of this methodology that takes advantage of existing software is also discussed. The methodology is demonstrated on a case study comparing the life-cycle impacts of two dialkylimidazolium-based ionic liquids,^37^ namely 1-butyl-3-methylimidazolium tetrafluoroborate [BMIM][BF_4_] and 1-butyl-3-methylimidazolium hexafluorophosphate [BMIM][PF_6_]. While [BMIM][BF_4_] and [BMIM][PF_6_] are not the most sustainable of ionic liquids available and their fluorine-based anions can hydrolyze under certain conditions,^38^ they have been used extensively for a range of applications^39−41^ and are thus of significant practical relevance. Since ionic liquid production processes are still at a low TRL, detailed process simulation becomes essential to assess their production at scale, and it becomes important to account for the foreground data uncertainty as a result.

## Methodology

The adopted LCA framework follows the four phases defined in the ISO 14040 standards: (i) goal and scope, (ii) inventory analysis, (iii) impact assessment, and (iv) interpretation. Choices about the system, including the scope, the boundaries of the foreground system, and the functional unit, are made in the goal and scope phase. Environmental flows for all inputs and outputs of each process in the complete process tree are collected during the life-cycle inventory (LCI) phase, including raw materials, energy streams, emissions, and wastes. Both the foreground and background inventories are then translated into environmental impacts during the life-cycle impact assessment (LCIA) phase through a characterization method, which is based on scientifically agreed environmental mechanisms with cause-effect pathways through which substances in emissions released or resources used can cause environmental damages. Lastly, the interpretation phase checks that the conclusions from the impact assessment are well-substantiated prior to making recommendations, and this is where uncertainty quantification and sensitivity analysis are applied.

Uncertainties in LCA stem from two main sources:^36^ (i) uncertain inventory flows into and out of processes within the technosphere or between processes and the ecosphere and (ii) uncertain characterization factors linking the ecosphere flows to environmental damages. Given the emphasis on emerging technologies, the main focus is on those uncertainties arising through the life-cycle inventories, which are further distinguished as foreground and background uncertainties subsequently. The former refer to uncertainties affecting the low TRL processes in the foreground system, where detailed process models are used to circumvent the gap in inventory data from state-of-the-art environmental databases, such as ecoinvent.^2^ These uncertain parameters include operating conditions, thermodynamic and physical properties, separation yields, and reaction rates, which translate to uncertainties on the flows exchanged between the foreground system and the rest of the technosphere or with the ecosphere. By contrast, background uncertainties are linked to the background processes and the supply chain activities, translating to further uncertainties on the flows between processes in the technosphere and with the ecosphere.

**Figure 1:**
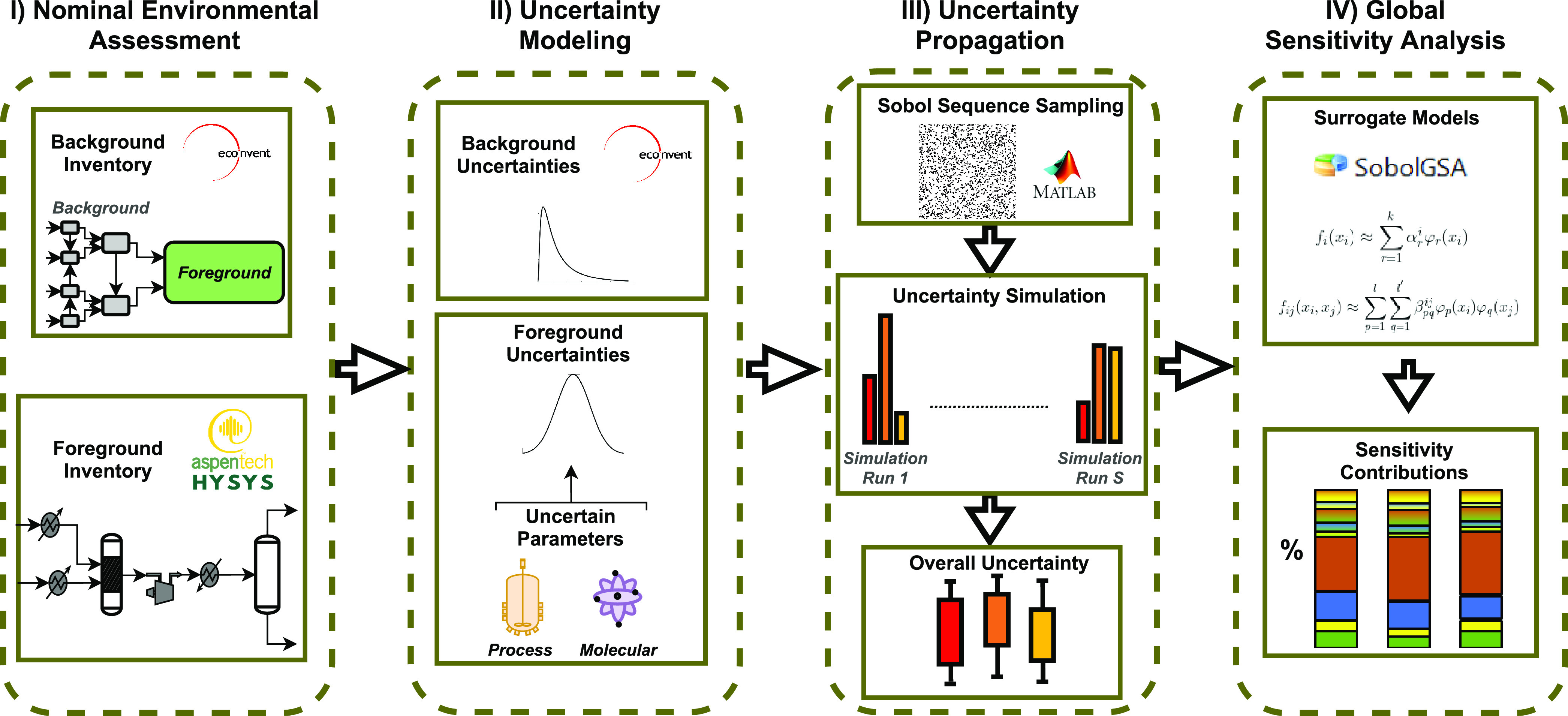
Methodology conceptual framework.

The proposed methodology for uncertainty propagation and analysis in LCA is summarized in Figure 1. Given the emphasis on emerging technology, a key novelty entails the combination of both background and foreground inventory uncertainties on the predicted environmental impacts. The framework starts with a nominal environmental assessment (Step I) combining LCA database information where available (e.g., background processes) with detailed process modeling to bridge inventory gaps (e.g., foreground processes). The next two steps entail characterizing and modeling the background and foreground uncertainties (Step II) before discretizing and propagating these uncertainties using (quasi) Monte Carlo sampling techniques (Step III), where each uncertainty realization is propagated through both the background and foreground inventories, and ultimately to the environmental impacts. The resulting impact uncertainty ranges are apportioned back to individual background and foreground uncertain factors as sensitivity indices (Step IV), using surrogate models trained on the sampled uncertainty scenarios to drive a variance-based GSA. Another key novelty here entails lumping multiple background inventories to reduce the dimensionality and improve the tractability of GSA in this context. The following subsections provide further details about the main steps.

### Modeling of Foreground and Background Life-Cycle Inventories

The overall environmental impact EI_*z*_ in a category *z* ∈ *Z* is determined using eq 1, expressed in units of impact per functional unit (FU). LCI_*e*_^tot^ denoftes the total life-cycle inventory of an elementary flow *e* ∈ *E* that is either consumed by a process within the technosphere or released by a process to the ecosphere, with units of elementary flow per FU. CF_*e*,*z*_ is the characterization factor of the elementary flow *e* in impact category *z*, with units of impact per elementary flow.
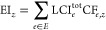
1

**Figure 2:**
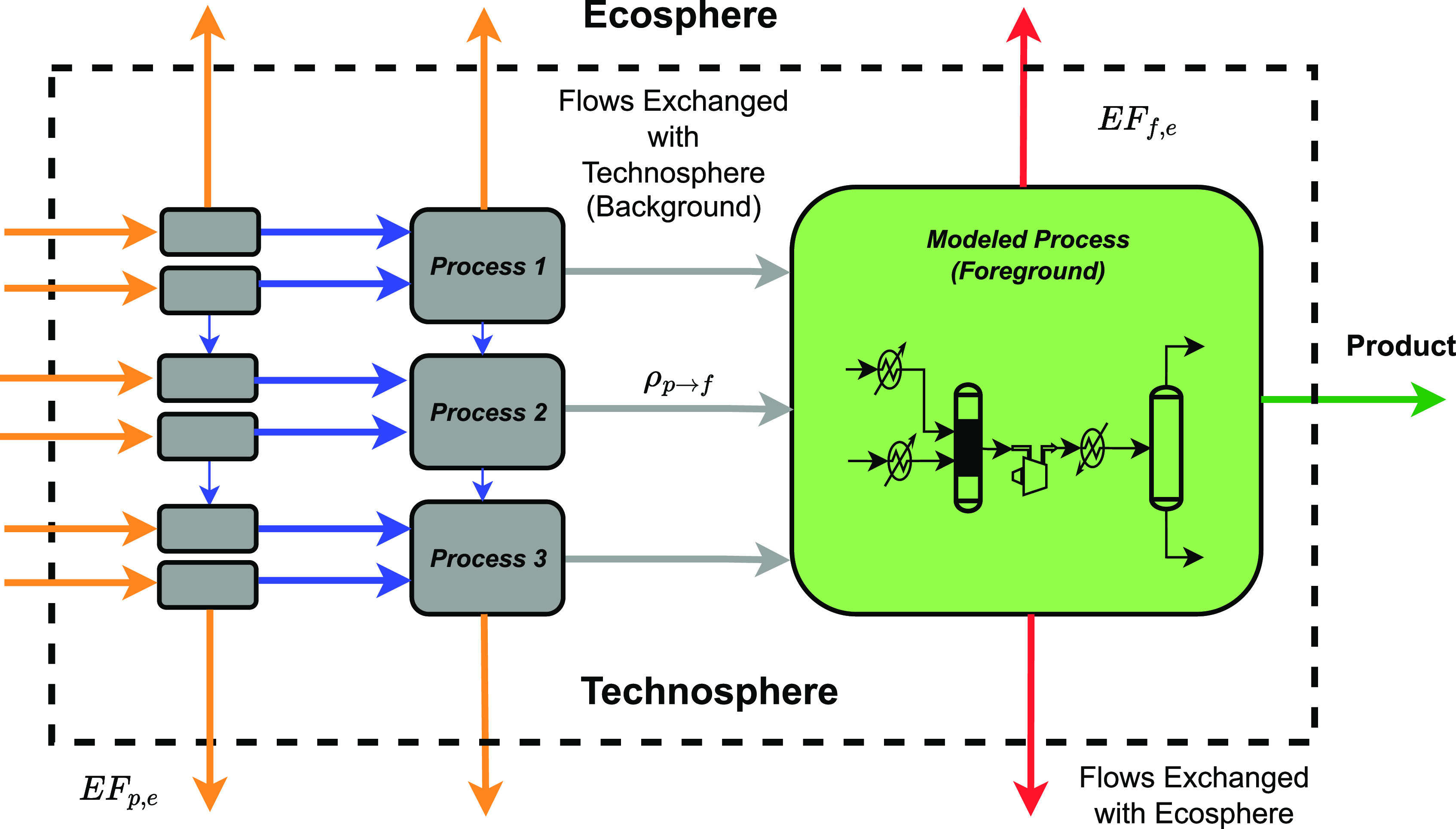
Conceptual diagram of a cradle-to-gate inventory illustrating the flows linking the foreground and background processes within the technosphere and with the ecosphere. The green arrow indicates the main product’s flow out of the foreground process, here assuming a single product. The red arrows show elementary flows EF_f,*e*_ exchanged between the foreground process and the ecosphere, while the orange arrows indicate elementary flows EF_*p*,*e*_ between the background process and the ecosphere. The intermediate flows shown with gray arrows are those exchanged between the foreground process and background processes located immediately upstream in the technosphere, and those with blue arrow are the intermediate flows between background processes in the technosphere. A cradle-to-grave LCA could be depicted similarly by including the background processes downstream of the foreground process.

In particular, LCI_*e*_^tot^ encompasses all the elementary flows in a reference product’s life cycle, including those exchanged between the foreground processes and the ecosphere and between the background processes and the ecosphere. For illustration, the diagram on Figure 2 depicts a cradle-to-gate LCI, where the foreground process exchanges elementary flows both with the ecosphere and with several background processes in the technosphere. This distinction between foreground and background processes is reflected in eq 2. There, EF_f,*e*_ denotes the elementary flow *e* exchanged between the foreground processes (indexed with f) and the ecosphere, in the same units as LCI_*e*_^tot^.  and  are the sets of processes immediately upstream and downstream of the foreground process, respectively. LCI_*p*,*e*_^up^ denotes the total inventory of elementary flow *e* from an immediate upstream process  and all the processes upstream of *p* in the process tree, with units of elementary flow per reference flow of mass or energy in process *p*, while using the factor ρ_*p*→f_ to rescale the elementary flow LCI_*p*,*e*_^up^ in terms of FU. Likewise, LCI_*p*,*e*_^down^ denotes the total inventory of elementary flow *e* from an immediate downstream process , with the factor ρ_f←*p*′_ rescaling  per FU.
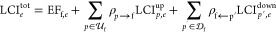
2

In turn, the total upstream inventory LCI_*p*,*e*_^up^ depends on the elementary flows EF_*p*,*e*_ from process *p* and EF_*p*′,*e*_ from all the processes *p*′ upstream of *p* within the technosphere, as well as all intermediate flows between any two background processes upstream of *p*. The total downstream inventory  has similar dependencies.

### Foreground and Background Uncertainty Quantification

For those background processes in which inventories are available in state-of-the-art LCI databases such as ecoinvent, the uncertainty quantification follows the Pedigree matrix approach,^24,42^ where the data sources are assessed according to the six characteristics of reliability, completeness, temporal correlation, geographic correlation, further technological correlation, and sample size, in addition to relying on expert judgments. For each uncertain elementary or intermediate flow (reference product flow excepted), a set of six indicator scores *U*^*c*^ is considered. These scores are combined with a basic uncertainty factor *U*^0^ to determine the standard deviation of a log-normal distribution for the corresponding flow. For instance, the case of an uncertain elementary flow EF_*p*,*e*_ is reported in eqs 3–4, where EF_*p*,*e*_^nom^ is the nominal value of the elementary flow (determined in Step I of the methodology, see Figure 1).
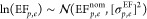
3
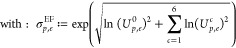
4

By contrast, for both the foreground processes and those background processes that are unavailable in state-of-the-art LCI databases, the main sources of uncertainty need to be characterized on a case-by-case basis. Detailed process models are developed to bridge such inventory gaps, and a key assumption henceforth is that the uncertainty can be described as uncertain parameter values in those models. These uncertain model parameters may either be linked to experimental errors in lab-scale procedures or inferred from expert opinions, e.g., when process scale-up is involved. Such knowledge informs the choice of a probability distribution for each parameter, including their shape, mean value, variance, and support set. One can further distinguish uncertain parameters corresponding to operating conditions that may be adjusted to mitigate impacts using process optimization (such as temperatures and pressures in unit operations), from uncertain physical parameters whose variation ranges may be refined through dedicated experiments or predictive ab initio simulations (including thermophysical properties, separation yields, reaction rates, and conversions).

The uncertain background flows are collectively denoted with the vector **φ** below, and the uncertain foreground parameters with the vector **ω**. In reference to eq 2, all of the elementary flows EF_f,*e*_ exchanged between the foreground processes and the ecosphere as well as the scaling factors ρ_*p*→f_ and ρ_f←*p*′_ directly depend on the foreground uncertainty realization **ω**, whereas the total upstream and downstream inventories LCI_*p*,*e*_^up^ and  depend on the background uncertainty realization **φ**.

The uncertainty scenario generation and propagation are coordinated from Matlab. They rely on a discretization of the foreground and background uncertainty (**ω**, **φ**) into a set of uncertainty scenarios through quasi-random (Sobol) sampling of their probability distributions. The proposed implementation proceeds by first simulating the foreground process flowsheets using Aspen-HYSYS interfaced with Matlab for each realization of **ω**, resulting in the foreground elementary flows EF_f,*e*_(**ω**) and the scaling factors ρ_*p*→f_(**ω**) and ρ_f←*p*′_(**ω**). Next, the elementary and intermediate flows in the background system are computed for the corresponding uncertainty realizations of **φ** using the database ecoinvent also interfaced with Matlab. This may entail simulating other process flowsheets developed for bridging gaps in the background inventories as well. All these flows are then combined into the total upstream and downstream inventories LCI_*p*,*e*_^up^(**φ**) and LCI_*p*,*e*_^down^(**φ**). Finally, these background inventories are rescaled and combined with the corresponding elementary flows EF_f,*e*_ from the foreground processes (eq 2), before applying a characterization method in Matlab to determine the predicted impacts EI_*z*_ in the mid-point or end-point categories of interest for each uncertainty scenario (eq 1). The relative error ϵ on the sample mean  for a sample size *N* at a given confidence level (1 – α)100% is estimated using eq 5, where  is the corresponding sample standard deviation.^43^ This estimate could also be used as a termination condition inside a loop that would increase the number of uncertainty scenarios incrementally.
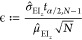
5

### Sensitivity Analysis of Foreground and Background Uncertainties

Analyzing the sensitivity of each environmental impact EI_*z*_ with respect to both the uncertain foreground parameters **ω** and background parameters **φ** entails quantifying the contribution of each of these parameters to the total variance of EI_*z*_. A key challenge in doing so is the presence of interactions between multiple uncertain parameters, so the total variance of EI_*z*_ may not be explained by simply adding up separate contributions from each parameter. Such interactions are evident from eq 2, where LCI_*p*,*e*_^up^(**φ**) and LCI_*p*^′^,*e*_^down^(φ) are respectively multiplied by ρ_*p*→f_(**ω**) and ρ_f←*p*′_(**ω**). Additional interactions may occur between the uncertain foreground parameters as well. Clearly, one-at-a-time sensitivity analysis is inappropriate in this context as it ignores such interactions, so one needs to resort to global sensitivity analysis (GSA) instead. The focus herein is on variance-based GSA techniques, which compute so-called Sobol indices that can be directly interpreted as measures of sensitivity. This class of GSA techniques are attractive because they measure sensitivity across the whole input space and compare favorably to other GSA approaches,^44^ yet they have not been widely applied in LCA applications thus far.^19^

A second challenge with analyzing the sensitivity of the environmental impacts EI_*z*_ is the high-dimensionality of the uncertain parameters **φ** in the background system. Herein, we propose to reduce this high-dimensionality by lumping multiple background parameters, as shown in eq 6. The new parameters BEI_*p*,*z*_^up^ (eq 7) represent the background environmental impact in category *z* generated by the immediate upstream process , either directly or via the processes upstream of *p* in the technosphere; the new parameters BEI_*p*^′^,*z*_^down^ (eq 8) have a similar interpretation for the immediate downstream process . For each impact category *z*, the size of these two sets of lumped parameters thus corresponds to the number of processes immediately upstream or downstream of the foreground processes times, a much smaller number compared to all the elementary and intermediate flows in the background system. Naturally, a follow-up sensitivity analysis can be conducted for any lumped parameter BEI_*p*,*z*_^up^ or BEI_*p*^′^,*z*_^down^ to identify its main contributing factors, and so on.
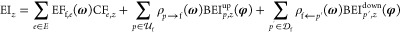
6
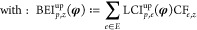
7
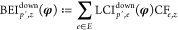
8

The implementation of variance-based GSA leverages the results of the joint foreground-background uncertainty propagation (Step III). The computation of the Sobol indices is conducted using the software SobolGSA,^45,46^ where the following indirect approach is selected. In the first step, metamodels are regressed for each impact EI_*z*_ with respect to the foreground uncertainties **ω** and the lumped background uncertainties BEI_*p*,*z*_^up^, BEI_*p*^′^,*z*_^down^, by leveraging the available samples from the foreground-background uncertainty propagation (Step III). The metamodel representation of choice is the random-sampling high-dimensional model representation (RS-HDMR).^47,48^ This representation is truncated to second-order interaction terms herein, which is sufficient to describe any binary interactions between a foreground and a background parameter, as well as binary interactions between two foreground parameters; only higher-order interactions between three or more parameters are thus neglected. In the second step, the coefficients of the RS-HDMR metamodel are used to compute the Sobol sensitivity indices^49^ at no additional cost. These sensitivity indices measure how much of the total variance of EI_*z*_ is attributable to the uncertain parameters, either separately (first-order effects) or to parameter pairs (second-order effects). It is worth noting that SobolGSA implements other metamodeling techniques and GSA approaches, which is convenient for verification and comparison purposes.

## Case Study Definition and Implementation

The proposed case study compares the environmental impacts associated with the production at scale of two dialkyl-imidazolium ionic liquids: 1-butyl-3-methylimidazolium tetrafluoroborate [BMIM][BF_4_] and 1-butyl-3-methylimidazolium hexafluorophosphate [BMIM][PF_6_]. Process flowsheeting in Aspen-HYSYS (version 9) is used to scale-up experimental synthesis procedures for [BMIM][BF_4_] and [BMIM][PF_6_],^50^ which comprise the foreground system. Process flowsheeting is also used to bridge inventory gaps for two of their precursors in ecoinvent as part of the background system, namely, 1-butyl-3-methylimidazolium chloride [BMIM][Cl] and 1-chlorobutane. The relevant process models and the methods and tools used to conduct the LCA are described in the following subsections, after which the main steps of the uncertainty quantification are summarized.

### Modeling of Ionic Liquid Production Processes

#### [BMIM][BF_4_] and [BMIM][PF_6_] Production

**Figure 3:**
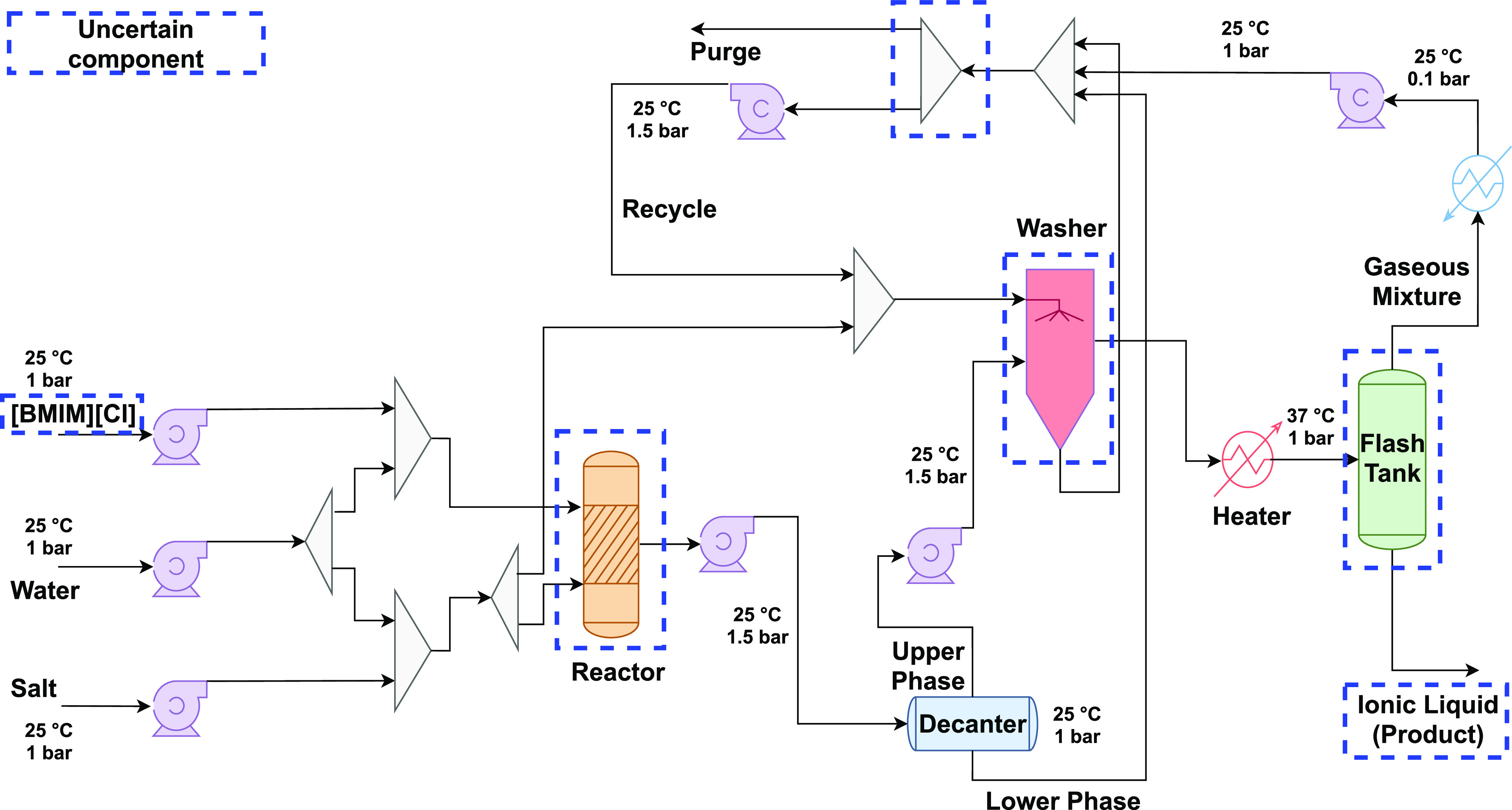
Process flow diagram of scale-up ionic liquid production. The dotted blue box indicates the unit operations with uncertain parameters.

The syntheses of [BMIM][BF_4_] and [BMIM][PF_6_] follow the metathesis procedure by Chen et al.^51^ (Figure 3). The paper by Chen et al.^51^ is among the few to provide sufficient details to enable modeling of the production of dialkylimidazolium-based ionic liquids at scale in order to predict the foreground inventories. For [BMIM][BF_4_], the synthesis proceeds via anion exchange between [BMIM][Cl] and sodium tetrafluoroborate NaBF_4_, producing solid sodium chloride NaCl as a byproduct—reaction R1 with X ≔ BF_4_ and Y ≔ Na. For [BMIM][PF_6_], the anion exchange is between [BMIM][Cl] and lithium hexafluorophosphate LiPF_6_—reaction R1 with X ≔ PF_6_ and Y ≔ Li.
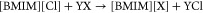
R1[BMIM][Cl] is mixed with an excess of YX under atmospheric conditions. The reaction mixture is separated into an upper phase, which contains the main aqueous product with impurities, and a lower phase containing solid YCl and undissolved YX. The upper phase is sent to a 3-stage washer using YX solution to remove impurities. In the final step, [BMIM][Y] is separated from water in a vacuum flash vessel, resulting in aqueous [BMIM][X] with 25 wt % water content.

#### [BMIM][Cl] Production

The production process of the [BMIM][Cl] precursor (Figure S1) is based on the experimental procedure reported by Baba et al.^52^ It starts by mixing 1-methylimidazole (NMIz) in toluene with excess 1-chlorobutane and running the reaction at 112 °C and under atmospheric pressure. [BMIM][Cl] is separated in a vacuum flash vessel from toluene and other unreacted materials which are returned to the reactor.

#### 1-Chlorobutane Production

The production process of 1-chlorobutane (Figure S2) starts by reacting 1-butanol with an excess of hydrogen chloride at 120 °C.^53^ The product mixture is cooled to 25 °C and sent to a first flash vessel tank, where the vapor phase containing mainly hydrogen chloride is separated. The liquid phase is then reheated to 69 °C and sent to a second flash vessel, where 1-chlorobutane is isolated from the residual 1-butanol and excess water.

#### Physical Property Estimation

UNIQUAQ is used as the thermodynamic package in Aspen-HYSYS. Since [BMIM][BF_4_], [BMIM][PF_6_], [BMIM][Cl], NaBF_4_, LiPF_6_, LiCl, and NMIz are currently unavailable in the Aspen-HYSYS database, pseudocomponents are created to estimate their properties. The methodology is described in Appendix A of the ESI, while the complete set of properties are reported in Tables S1–S7. Physical properties such as densities are retrieved from the literature.^54^ Critical properties and normal boiling points of the ionic liquids are estimated using the group contribution method by Valderrama and Rojas.^55^ Properties of other pseudo components are estimated from their molecular structure using the Aspen-HYSYS built-in property constant estimation system (PCES). Heat of formations are determined through quantum calculations.

### Environmental Assessment

The LCA follows the four phases of the ISO 14040 standards, as detailed below. The nominal LCA (phases ii and iii) is conducted using the software SimaPro (version 9) interfaced with ecoinvent 3.5.^2^ By contrast, the uncertainty analyze (phase (iv) is coordinated from Matlab in order to enable joint foreground and background uncertainty quantification, which is currently not possible with SimaPro.

#### i Goal and Scope

The goal of the environmental assessment is to compare the production of the dialkyl-imidazolium ionic liquids [BMIM][BF_4_] and [BMIM][PF_6_]. A cradle-to-gate scope is adopted, which includes all processes from raw material extraction to the ionic liquid production, but excludes any further processing, use or waste management after the production. Since ionic liquids are commonly sold by weight, the functional units is defined as “1 kg of ionic liquid”. It is furthermore assumed that [BMIM][BF_4_] and [BMIM][PF_6_] are the single products of each process alternatives, so no allocation is needed, and the geographical location is chosen as Europe.

#### ii Life-Cycle Inventory (LCI)

Mass and energy flows for the production processes of [BMIM][BF_4_] and [BMIM][PF_6_] and both precursors [BMIM][Cl] and 1-chlorobutane are predicted using process flowsheeting in Aspen-HYSYS. These inventories are combined with data gathered from ecoinvent for the rest of the background processes in order to quantify the life-cycle inventories of [BMIM][BF_4_] and [BMIM][BF_6_]. A complete list of the foreground inventory flows, expressed for the functional unit, can be found in Tables S9–S12. The methods used to quantify the air and water emissions are reported in Table S8. They follow the guidelines by Hischier et al.,^56^ which are used for many processes in ecoinvent and ensure consistency.

#### iii Life-Cycle Impact Assessment (LCIA)

The LCI entries are converted into environmental impacts using the ReCiPe 2016 methodology.^57^ These impacts are first categorized into 18 mid-point indicators, including global warming, toxicity, ozone depletion and land use, and then further aggregated into three end-point categories: the damage areas of resources, human health, and ecosystems quality. The assessment follows the hierarchist perspective, which is based on the cultural theory of scientific agreement and adopts a medium time frame of 100 years for the environmental impacts. The complete ReCiPe mid-point and end-point results are given in Tables S13 and S14, respectively, for the functional unit.

#### iv Interpretation and Uncertainty Analysis

The quantification of both foreground and background uncertainties follows the proposed methodology (Steps II to IV in Figure 1). In the foreground system, nine uncertain parameters are considered in the process models of [BMIM][BF_4_] and [BMIM][PF_6_] production (cf., Tables 1 and S15). Five of them correspond to uncertain operating conditions, namely, the pressure drops in the reactor (Δ*P*_R_) and in the washer (Δ*P*_W_), the temperature (*T*_VF_) and pressure (*P*_VF_) in the vacuum flash vessel, and the purge split ratio (PUR); cf., Figure 3 where the corresponding units are identified. Most of these operating conditions (Δ*P*_R_, Δ*P*_W_, *P*_VF_, PUR) are highly uncertain since the process models are scale-up from experimental synthesis procedures and thus described by a triangular distribution with a range of wide ±50% around their nominal values; a smaller uncertainty range of ±20% is considered for the operating temperature *T*_VF_ in the vacuum flash unit as the nominal temperature corresponds to the maximal product yield and temperature can easily be controlled around this value in practice. The remaining four uncertain parameters correspond to thermophysical properties, namely the heats of formation and densities of [BMIM][BF_4_], [BMIM][PF_6_], and [BMIM][Cl]. These uncertainties are also modeled using triangular distributions, with nominal values and uncertainty ranges based on experimental errors from the literature. Concerning the background system, parametric uncertainties are considered in the process models of [BMIM][Cl] and 1-chlorobutane production in the same way. These uncertain parameters are reported in Tables S16 and S17 with their corresponding nominal values and uncertainty ranges for completeness. Sample generation for all these uncertain parameters is coordinated from Matlab using quasi Monte Carlo sampling based on low-discrepancy Sobol sequences^58^ and interfaced with Aspen-HYSYS for simulating the process flowsheets in each uncertainty scenario. As explained in the methodology section, these are combined with uncertainty scenarios of the elementary and intermediate background flows in order to predict the distribution of each environmental impact EI_*z*_. A total of 10,000 uncertainty scenarios are used for the various cases discussed in the following section. The relative error ϵ on the mean of each environmental impact, estimated using eq 5 at a 95% confidence level, is in the range between 0.04–0.05. Further details about the implementation can be found in Appendix E of the ESI.

**Table 1:** Uncertain Model Parameters, Uncertainty Sources, and Ranges in Flowsheet Simulation of [BMIM][BF_4_] Production^a^

Type	Parameter	Range	Units
Operating condition	Δ*P*_R_^b^	10 ± 50%	kPa
	Δ*P*_W_^b^	10 ± 50%	kPa
	*T*_VF_^c^	80 ± 20%	°C
	*P*_VF_^b^	10 ± 50%	kPa
	PUR^b^	0.1 ± 50%	-
			
Thermophysical property	ρ_[BMIM][BF_4_]_^d^	1208 ± 19%	kg m^–3^
	ρ_[BMIM][Cl]_^d^	1080 ± 19%	kg m^–3^
	Δ*H*_f__[BMIM][BF_4_]_^e^	–6.50 ± 1.59 × 10^5^	kJ kmol^–1^
	Δ*H*_f__[BMIM][Cl]_^e^	–2.37 ± 1.59 × 10^5^	kJ kmol^–1^

a Each uncertain parameter is assumed to follow a triangular distribution.

b Estimate based on heuristics.

c Mean value based on an optimized base case.

d Estimate based on the group contribution methods developed by Valderrama and Rojas^55^ with maximum standard deviation of 19%.

e Estimate based on the lattice energy and computational chemistry methods proposed by Gao et al.^59^ with maximum deviation of −159 kJ mol^–1^.

In the sensitivity analysis following the uncertainty propagation (Step IV), the following seven lumped background impacts are considered alongside the nine uncertain foreground parameters: production of [BMIM][Cl] (BEI_*z*_^[BMIM][Cl]^), production of sodium tetrafluoroborate (BEI_*z*_^NaBF_4_^) or lithium hexafluorophosphate (BEI_*z*_^LiPF_6_^), production of construction materials (BEI_*z*_^mat^), production of thermal energy (BEI_*z*_^th^), production of electricity (BEI_*z*_^el^), production of water (BEI_*z*_^wat^), and wastewater treatment (BEI_*z*_^wwt^). Recall that the uncertainty realizations for all these lumped background impacts can be computed using the elementary and intermediate background flow samples that are already available from the uncertain propagation (Step III, eqs 7 and 8). But since the lumped background impacts are specific to a particular impact category, a separate GSA needs to be conducted for each impact category *z*. Of the 10,000 samples available from the uncertainty propagation, 9,000 are used to construct the RS-HDMR metamodels in SobolGSA and the remaining 1,000 samples are used for testing. The coefficients of the RS-HDMR metamodels are estimated via regression. The statistical fitness measure for the metamodels of different end-point impact categories for both ionic liquids is *R*^2^ > 0.90. Finally, the Sobol indices derived from the RS-HDMR model coefficients are normalized by the sample variance of the corresponding impact EI_*z*_ (rather than the sum of the first- and second-order indices) in order to detect the presence of higher-order interactions.

## Case Study Results and Discussions

### Nominal Environmental Assessment

**Figure 4:**
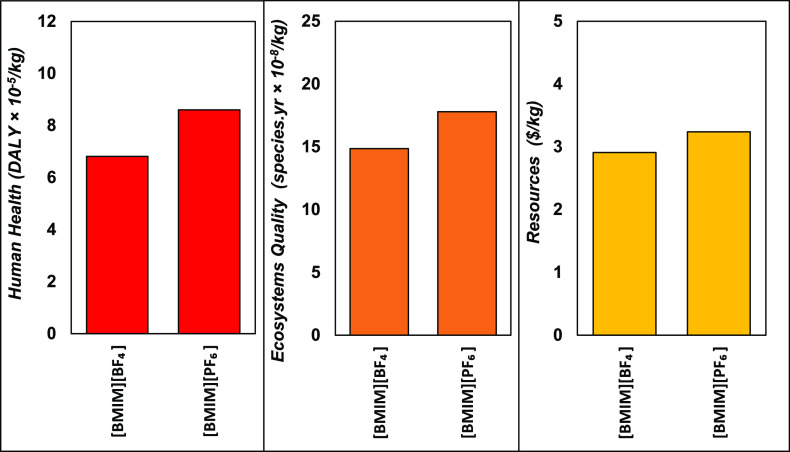
Nominal LCA comparison of end-point indicators for the production of [BMIM][BF_4_] and [BMIM][PF_6_].

The bar charts in Figure 4 summarize the nominal LCA results for all three end-point damage categories—human health, ecosystems quality and resources—on a per-weight basis of ionic liquid. The complete set of mid-point and end-point indicators can be found in Tables S13 and S14 of the ESI. This nominal comparison suggests that the production of [BMIM][BF_4_] presents lower environmental impacts than [BMIM][PF_6_] in all damage areas. Damages on human health are reduced by 21%, on ecosystems quality damage by 16%, and on resources by 10%. Since both ionic liquids are produced using the same process and share the same cation, these differences are attributed to the different anions used and their respective production trees.

Under the damage area of human health, producing the precursor [BMIM][Cl] contributes, respectively, 32% and 20% of the life-cycle impacts of [BMIM][BF_4_] and [BMIM][PF_6_]. This is significantly less than the production of their anionic counterparts NaBF_4_ and LiPF_6_, which contribute 65% and 79%, respectively. The largest mid-point contributions to this end-point damage area for both ionic liquids are global warming, mostly due to carbon dioxide emissions; and fine particulate formation, mainly due to emissions of sulfur dioxide and <2.5 μm particulate matter.

Under the area of ecosystems quality, [BMIM][Cl] production is responsible for, respectively, 45% and 29% of the impacts of [BMIM][BF_4_] and [BMIM][PF_6_], while their anionic counterparts NaBF_4_ and LiPF_6_ again contribute a larger share of 51% and 69%. The main mid-point contributions to this end-point damage area for both ionic liquids are global warming (>50%), acidification, terrestrial ozone formation, and water consumption. Acidification is mainly due to sulfur dioxide emissions, ozone formation to toluene emissions, and water consumption to hydropower electricity production.

Under the resources area, the production of [BMIM][Cl] is responsible for a majority (56%) of the impacts of [BMIM][BF_4_] followed by the production of NaBF_4_ (42%). These contributions are flipped for [BMIM][PF_6_] with the production of LiPF_6_ causing a majority of the impacts (61%) compared to the production of [BMIM][Cl] (38%). Part of this difference is explained by the fact that PF_6_ is heavier than BF_4_, making 51% of molecular weight of [BMIM][PF_6_], while BF_4_ only makes 38% of the molecular weight of [BMIM][BF_4_]. Nearly all of these end-point damages are caused by fossil resource scarcity (>99%) at the mid-point level, mainly due to natural gas (>45%) and crude oil (>45%) used by the various processes or for the transportation of intermediates.

At this point, it is worth noting that the values of several mid-point indicators for [BMIM][BF_4_] production differ widely from those predicted by Zhang et al.^11^ For instance, the predicted global warming impact (27.3 kġCO_2_-eq kg^–1^˙[BMIM][BF_4_], cf., Table S13) is an order of magnitude higher than the impact reported by Zhang et al.^11^ (3.5 kġCO_2_-eq kg^–1^˙[BMIM][BF_4_]). This is mainly due to the latter relying on stoichiometric calculations and other simplifying modeling assumption for the production of both the ionic liquids and their precursors, which do not account for reaction yields, heating and cooling requirements, separation efficiency, and waste and emissions. Hence, the LCA results presented herein can be considered more reliable.

### Effect of the Foreground and Background Uncertainties

**Figure 5:**
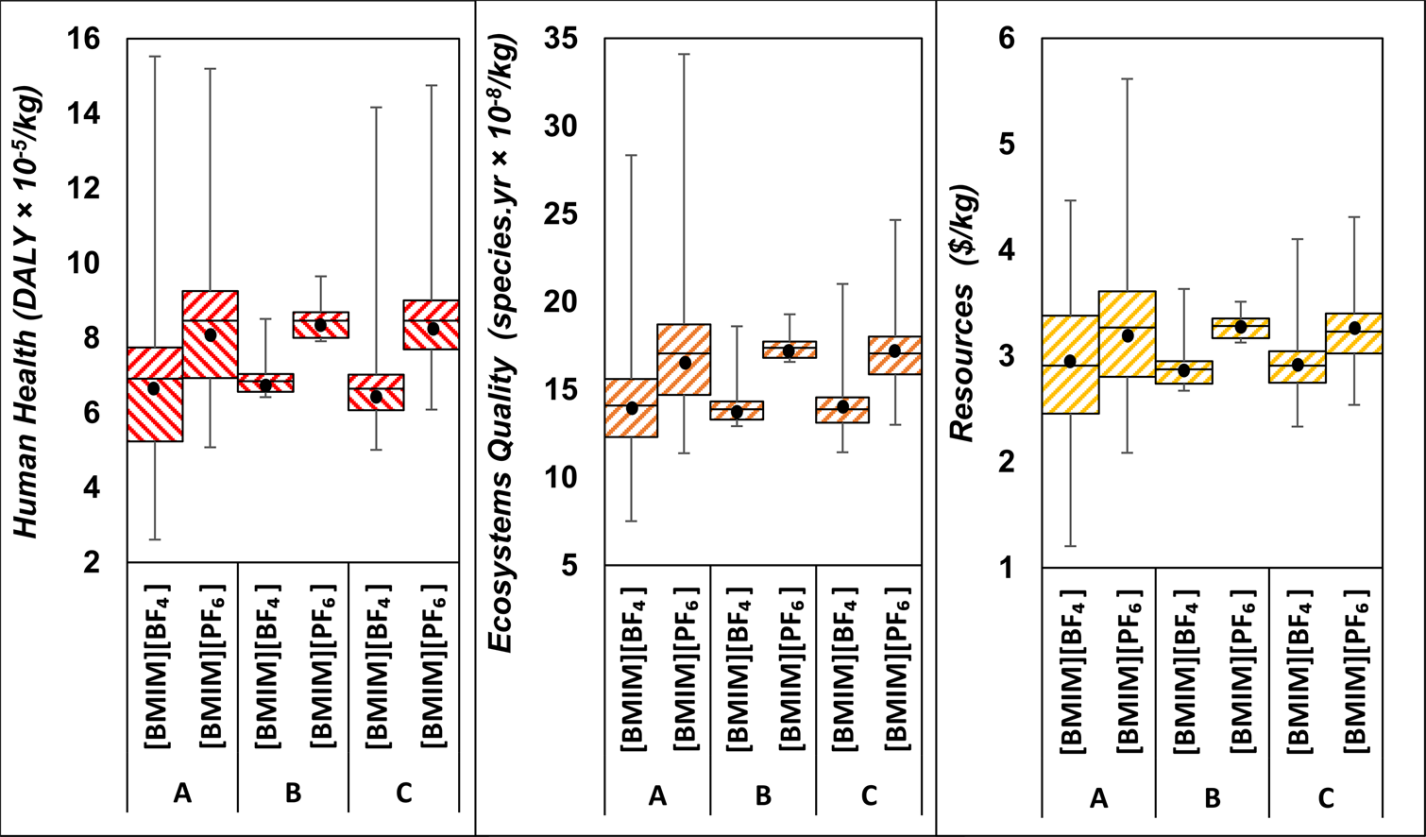
LCA comparison of end-point indicators for the production of [BMIM][BF_4_] and [BMIM][PF_6_] under combined foreground/background uncertainty (A), foreground uncertainty only (B), and background uncertainty only (C). A total of 10,000 uncertainty scenarios are used in each case. The black points represent the mean scenario; the central line inside each box represents the median scenario; the lower and upper ends of the box represent the first and third quartiles, respectively; and the lower and upper extended lines of the box represent the minimum and maximum values, respectively.

Comparing both ionic liquids in terms of their nominal LCA performance could lead to believing that the production of [BMIM][BF_4_] presents lower environmental impacts than [BMIM][PF_6_] in all damage areas and therefore discard the latter. However, the box plots in Figure 5 depict a different reality, whereby the range of impacts of both ionic liquids overlap significantly.

When all the foreground and background uncertainties are considered simultaneously (scenario A), the damages caused by [BMIM][BF_4_] on human health (left plot), ecosystems quality (middle plot), and resources (right plot) are higher than those caused by [BMIM][PF_6_] in 21%, 15%, and 29% of the uncertainty scenarios, respectively (cf., top plot of Figure S3). This overlap is significantly larger than under the traditional approach of considering solely the background uncertainties (scenario C), where the damages caused by [BMIM][BF_4_] on human health, ecosystems quality, and resources are higher than those of [BMIM][PF_6_] in 8%, 5%, and 20% of the scenarios only (cf., middle plot of Figure S3). Clearly, adding the foreground uncertainty to the background uncertainty (scenario A) is necessary for a more reliable comparative assessment of these two ionic liquids.

When considering the foreground uncertainties alone (scenario B), notice that [BMIM][BF_4_] presents lower impacts on human health, ecosystems quality and resources in nearly all of the uncertainty scenarios. But even though the effect of the foreground uncertainties appears to be modest in comparison to that of the background uncertainties, the combined effect of the foreground and background uncertainties is significantly larger, with interquartile ranges about twice greater for the environmental impacts in scenario A compared to scenario C. This is mainly due to the multiplicative effect between foreground and background uncertainties, as illustrated in eq 6.

### Global Sensitivity Analysis of the Impact Assessment

**Figure 6:**
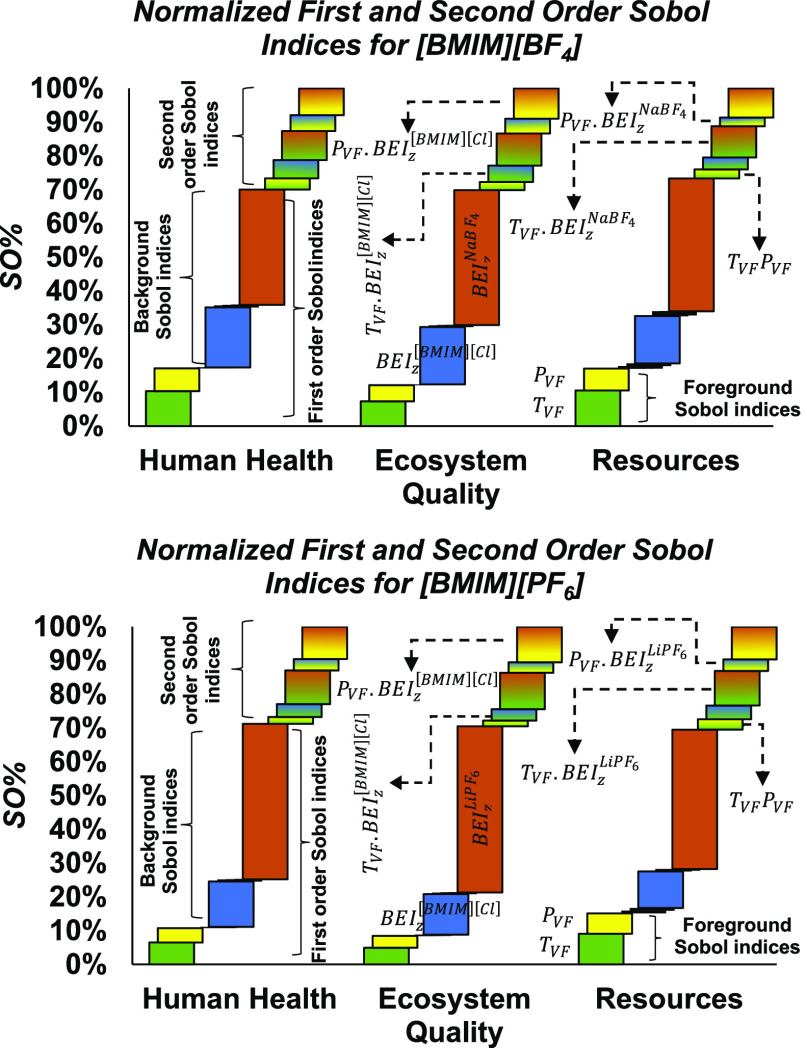
Breakdown of the sampled variance of each end-point impact EI_*z*_ in terms of their first- and second-order Sobol indices for [BMIM][BF_4_] (top) and [BMIM][PF_6_] (bottom).

The bar charts in Figure 6 show a breakdown of the sampled variance of each end-point impact EI_*z*_ in terms of their first- and second-order Sobol indices, for both ionic liquid production processes and under combined foreground/background uncertainty. The complete set of Sobol indices can be found in Tables S18 and S19 of the ESI. It is found that a majority of the end-point impact variance is attributable to the background uncertainties, which is in agreement with the comparison between scenarios B and C in Figure 5. In the case of the ecosystems quality impact of [BMIM][PF_6_] for instance, the first-order effects of the background uncertainty add up to 62%, while the combined first-order effects of the foreground uncertainty are only 9%. Specifically, the most sensitive background lumped parameters correspond to the production of the metal salt precursors NaBF_4_ and LiPF_6_ used, respectively, for synthesizing [BMIM][BF_4_] and [BMIM][PF_6_], and to a lower extend the precursor ionic liquid [BMIM][Cl]. This is not surprising insofar as the production of these precursors involves a number of complex synthesis steps, some of which featuring highly uncertain parameters, in comparison to other established background activities such as thermal energy and electricity production. With regards to the foreground uncertainty, by far the most sensitive process parameters correspond to the temperature *T*_VF_ and pressure *P*_VF_ in the vacuum flash vessel. The former impacts the evaporative (trace) losses of ionic liquid, whereas the latter modifies the phase equilibrium of the ionic liquid-water mixture, which are both impacting the final yields of ionic liquid. On top of these first-order effects, second-order interactions between the foreground and background parameters also contribute significantly (around 30%) to the variance of the environmental impacts while higher-order interactions are negligible in this case. Such second-order effects were indeed expected given that the variance in scenario A of Figure 5 is much larger than those of scenarios B and C combined. The largest interactions are between the lumped background parameters BEI_*z*_^[BMIM][Cl]^, BEI_*z*_^NaBF_4_^, or BEI_*z*_^LiPF_6_^ and the vacuum flash temperature *T*_VF_ and pressure *P*_VF_ in the foreground processes. A small interaction is also observed between the two foreground parameters *T*_VF_ and *P*_VF_ due to their joint effect on the phase equilibrium of the ionic liquid-water mixture, whereas no interaction between the lumped background parameters is permitted here due to their additive structure in eq 6. At this stage, it is worth noting that since the foreground uncertainty parameters correspond to the same operational uncertainties in both production processes of [BMIM][BF_4_] and [BMIM][PF_6_], they would likely be set in a consistent way in industrial processes. Therefore, it might be conservative to treat these uncertainties as independent.

**Figure 7:**
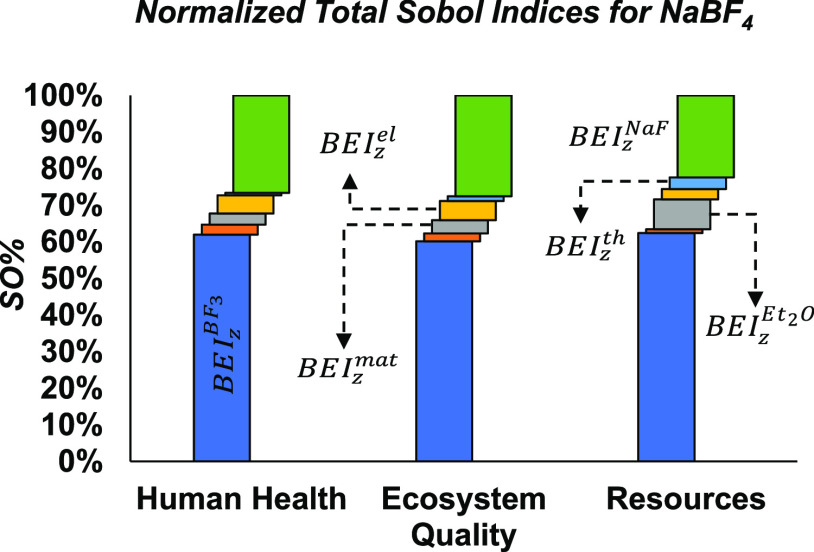
Breakdown of the sampled variance of the lumped background parameters BEI_*z*_^NaBF_4_^ in all three end-point impact categories in terms of Sobol indices, under combined foreground/background uncertainty.

Given the prominent role of the ionic liquid precursors on the variance of the environmental impacts, a follow-up GSA is worth conducting to further apportion the uncertainty between the lumped background parameters BEI_*z*_^[BMIM][Cl]^, BEI_*z*_^NaBF_4_^, and BEI_*z*_^LiPF_6_^, now acting as outputs, in terms of their upstream process activities. To exemplify this process, the bar charts in Figure 7 show a breakdown of the sampled variance of BEI_*z*_^NaBF_4_^ in each end-point impact category *z*, where the corresponding lumped background activities are production of boron trifluoride (BEI_*z*_^BF_3_^), production of sodium fluoride (BEI_*z*_^NaF^), production of dethyl ether (BEI_*z*_^Et_2_O^), production of construction materials (BEI_*z*_^mat^), production of thermal energy (BEI_*z*_^th^), and production of electricity (BEI_*z*_^el^) . The production of the main reagents BF_3_ and NaF, both required in large quantities, accounts for most of the variance in this background parameter, namely, BEI_*z*_^BF_3_^ (58–60%) and BEI_*z*_^NaF^ (25–30%)—cf., Table S20 for the complete sensitivity results. In the resources damage area, diethly ether also contributes a non-negligible share (8%) of the variance of BEI_*z*_^NaBF_4_^ as this solvent is fossil-based and subject to large evaporative losses. By construction, the contributions of lumped background parameters BEI_*z*_^BF_3_^, BEI_*z*_^NaF^, BEI_*z*_^Et_2_O^, BEI_*z*_^mat^, BEI_*z*_^th^ ,and BEI_*z*_^el^ to BEI_z_^NaBF_4_^ are separable, so this apportionment only comprises first-order Sobol indices, no second-order effects. Similar conclusions can be drawn on apportioning the variance of BEI_*z*_^LiPF_6_^ between their upstream activities (cf., Tables S21).

Instead of applying variance-based GSA, other approaches such as a one-at-a-time sensitivity analysis (OTSA) could be pursued to analyze the LCA results. Applied to the foreground system, OTSA allows ranking of the uncertain foreground parameter in order of importance. One caveat with OTSA, however, is that it keeps all the parameters except one constant at their nominal values, thereby neglecting cross-interactions among parameters as well as nonlinearity effects for the set parameters. Using the same uncertainty ranges as in Table 1, it is found that OTSA dramatically underestimates the sensitivity of the temperature *T*_VF_ and pressure *P*_VF_ in the vacuum flash vessel compared to the other foreground parameters, in particular the purge split ratio whose sensitivity is greatly overestimated (cf., Table S18). This is because these two parameters present a strong mutual interaction and cause large variations when their values are low compared to the nominal temperature and pressure. Regarding the background system, one could apply a similar parameter lumping as in eq 6 to reduce the dimensionality of the sensitivity analysis. Nevertheless, the main limitation remains that OTSA cannot account for any of the interactions between the foreground and background parameters, which are known to contribute significantly to the variance of the environmental impacts (cf., Figures 5 and 6). This is in contrast to variance-based GSA methods that evaluate the effect of a parameter while also varying all the other parameters, thereby accounting for cross-interactions between parameters and being independent of the choice of a nominal point. The interpretation of the Sobol indices is furthermore unambiguous in terms of apportioning the variance of the environmental impacts to the foreground and background parameters. All this leads us to argue for variance-based GSA to become the method of choice in LCA of early-stage technology.

## Conclusions

This paper has presented a methodology for reliable life-cycle assessment of emerging technologies that relies on detailed process simulation to bridge the gaps in foreground or background inventory data. This methodology builds upon nominal LCA to quantify the environmental impacts in different damage areas and identify the activities contributing the most to these impacts. One main novelty herein entails quantifying the variance of the environmental impacts via joint uncertainty propagation in the foreground and background inventories, including for the first time uncertain physical parameters and uncertain design or operating parameters in the process models used to predict the performance of early-stage technology at scale. A second key contribution is a tailored GSA approach to apportioning the variance of each environmental impact in terms of the foreground and background uncertainties, whereby a reduced set of lumped background parameters corresponding to the immediate upstream and downstream processes is considered rather than the whole lot of uncertain background flows. This lumping facilitates the sensitivity results interpretation, while not impairing the generality of the analysis since a follow-up GSA may be conducted for the most sensitive lumped parameters if necessary. And unlike traditional approaches such as one-at-a-time sensitivity analysis for ranking the uncertain parameters in order of importance, variance-based GSA measures sensitivity across the whole parameter space, including cross-interactions between parameters and making it intuitive to interpret the resulting Sobol indices in terms of variance contributions.

Another strong point of this methodology is that its implementation leverages state-of-the-art software, such as the process simulator Aspen-HYSYS with the database ecoinvent interfaced with Matlab and GSA toolkit SobolGSA, and may be conveniently orchestrated from a platform, such as Matlab. Although this may also be seen as a drawback of the methodology since it requires searching the database ecoinvent to trace the background processes, a step that is normally hidden from the user in LCA software such as SimaPro or OpenLCA. Nevertheless, once the interface between Aspen-HYSYS and ecoinvent has been built in Matlab, setting up a new case study would only entail assembling a flowsheet for the foreground processes and specifying the foreground uncertainties. Another challenge may be of computational nature as the foreground inventories need to be recomputed for each foreground uncertainty scenario, then propagated through the background system, which may prove time-consuming for a complex flowsheet.

Ionic liquid production, which remains at a low technology-readiness level, has provided the case study for demonstrating the methodology. A nominal LCA comparison between the dialkyl-imidazolium ionic liquids [BMIM][BF_4_] and [BMIM][PF_6_] showed that the former has lower environmental impacts by 10–20% in all three end-point damage areas and nearly all of these impacts are associated with the production of the precursors [BMIM][Cl], BF_4_, and PF_6_. However, a different reality emerged after quantifying the effect of both foreground and background uncertainties on the environmental impact predictions due to significant overlaps between the impact ranges of [BMIM][BF_4_] and [BMIM][PF_6_]. This analysis also revealed that the consideration of foreground uncertainty alongside the background uncertainty could about double the impact ranges compared to the effect of background uncertainty alone. The results of the variance-based GSA could then establish that a majority of the impact ranges are caused by only four uncertain parameters: the lumped background parameters representing the production impacts of the precursors [BMIM][Cl] and either BF_4_ or PF_6_—the variations of which are themselves attributed mainly to the production of solvents and reagents; and the vacuum flash temperature and pressure in the foreground processes. Significant interactions were also exposed between the foreground and background parameters, in agreement with the uncertainty quantification results. Overall, these findings illustrate how foreground uncertainties in early-stage technology assessment can exacerbate the variability of environmental impacts and therefore the need to quantify them.

Finally, this methodology could provide useful insight in guiding the selection and production of more sustainable ionic liquids, where the identification of key foreground uncertainties early on might help refocus research efforts. It could also be used in the assessment of early-stage technology beyond ionic liquids. Some of our current investigations there include CO_2_ utilization technology and other feedstock recycling technologies for certain types of plastics waste.

## Supporting Information Available

Nomenclature, property estimation and process flowsheeting, environmental assessment data and methodology, uncertainty analysis data and comparison, and uncertainty quantification methodology and results (PDF)
