# Supplementary material for: Global Sensitivity Analysis in Life-Cycle Assessment of Early-Stage Technology using Detailed Process Simulation: Application to Dialkylimidazolium Ionic Liquid Production
Source: ACS Sustain Chem Eng. 2023 Apr 21;11(18):7157–69. doi: 10.1021/acssuschemeng.3c00547 (PMC10170515; doi:10.1021/acssuschemeng.3c00547)
Supplement: Supplementary file 1 — sc3c00547_si_001.pdf [file sc3c00547_si_001.pdf]

ELECTRONIC SUPPLEMENTARY INFORMATION

Global Sensitivity Analysis in Life-Cycle Assessment of Early-Stage Technology using Detailed Process Simulation: Application to Dialkylimidazolium Ionic Liquid Production

Baaqel, Husain A.[1,2], Bernardi, Andrea[1,2], Hallett, Jason P.[1], Guillén-Gosálbez, Gonzalo[3], and Chachuat, Benoît *[1,2]

[1]Department of Chemical Engineering, Imperial College London, South Kensington Campus, London SW7 2AZ, United Kingdom
[2]Sargent Centre for Process Systems Engineering, Imperial College London, South Kensington Campus, London SW7 2AZ, United Kingdom
[3]Institute for Chemical and Bioengineering, Swiss Federal Institute of Technology, Vladimir-Prelog-Weg 1, Zurich 8093, Switzerland

Number of pages: 23
Number of figures: 3
Number of tables: 21

*Corresponding Author: b.chachuat@imperial.ac.uk

A. Nomenclature

Acronyms

FU	functional unit
GSA	global sensitivity analysis
LCA	life-cycle assessment
LCI	life-cycle inventory
LCIA	life-cycle impact assessment
OTSA	one-at-a-time sensitivity analysis
TRL	technology-readiness level

Chemicals

BF_4	tetrafluoroborate
BMIM	1-butyl-3-methylimidazolium
NMIz	1-methylimidazole
PF_6	hexafluorophosphate

Symbols

$\mathrm{BEI}_z^{\mathrm{BF_3}}$	lumped background impact of production of boron trifuoride
$\mathrm{BEI}_z^{\mathrm{[BMIM][Cl]}}$	lumped background impact of production of sodium tetrafluoroborate
$\mathrm{BEI}_z^{\mathrm{el}}$	lumped background impact of production of electricity
$\mathrm{BEI}_z^{\mathrm{Et_2O}}$	lumped background impact of production of dethyl ether
$\mathrm{BEI}_z^{\mathrm{LiPF_6}}$	lumped background impact of production of lithium hexafluorophosphate
$\mathrm{BEI}_z^{\mathrm{mat}}$	lumped background impact of production of construction materials
$\mathrm{BEI}_z^{\mathrm{NaBF_4}}$	lumped background impact of production of sodium tetrafluoroborate
$\mathrm{BEI}_z^{\mathrm{NaF}}$	lumped background impact of production of sodium fluoride
$\mathrm{BEI}_z^{\mathrm{th}}$	lumped background impact of production of thermal energy
$\mathrm{BEI}_z^{\mathrm{wat}}$	lumped background impact of production of water
$\mathrm{BEI}_z^{\mathrm{wwt}}$	lumped background impact of wastewater treatment
$\mathrm{CF}_{e,z}$	characterization factor of elementary flow $e \in E$ in impact category $z \in Z$
$\mathcal{D}_\mathrm{f}$	set of processes immediately downstream of foreground process f
$\mathrm{EF}_{\mathrm{f},e}$	elementary flow $e \in E$ exchanged between foreground process f and ecosphere
$\mathrm{EF}_{p,e}$	elementary flow $e \in E$ exchanged between background process p and ecosphere
$\mathrm{EF}_{p,e}^{\mathrm{nom}}$	nominal value of elementary flow elementary flow $\mathrm{EF}_{p,e}$
EI_z	overall environmental impact in category $z \in Z$
$\mathrm{LCI}_e^{\mathrm{tot}}$	total life-cycle inventory of elementary flow $e \in E$
$\mathrm{LCI}_{p,e}^{\mathrm{down}}$	total inventory of elementary flow $e \in E$ from immediate downstream process $p \in \mathcal{D}_\mathrm{f}$ and all processes downstream of p in process tree
$\mathrm{LCI}_{p,e}^{\mathrm{up}}$	total inventory of elementary flow $e \in E$ from immediate upstream process $p \in \mathcal{U}_\mathrm{f}$ and all processes upstream of p in process tree
N	sample size

PUR	purge split ratio
P_{VF}	pressure of vacuum flash vessel
T_{VF}	temperature of vacuum flash vessel
U^c	indicator score in Pedigree matrix approach
$\mathcal{U}_{\mathrm{f}}$	set of processes immediately upstream of foreground process f
$\Delta H_{\mathrm{f}\,[\mathrm{BMIM}][\mathrm{BF}_4]}$	heat of formation of $[\mathrm{BMIM}][\mathrm{BF}_4]$
$\Delta H_{\mathrm{f}\,[\mathrm{BMIM}][\mathrm{Cl}]}$	heat of formation of [BMIM][Cl]
ΔP_{R}	pressure drop in reactor
ΔP_{W}	pressure drop in washer
ϵ	relative error
$\hat{\mu}_{\mathrm{EI}_z}$	sample mean of environmental impact EI_z
$\rho_{[\mathrm{BMIM}][\mathrm{BF}_4]}$	density of $[\mathrm{BMIM}][\mathrm{BF}_4]$
$\rho_{[\mathrm{BMIM}][\mathrm{Cl}]}$	density of [BMIM][Cl]
$\rho_{\mathrm{f}\leftarrow p}$	scaling factor for elementary flow $\mathrm{LCI}^{\mathrm{down}}_{p,e}$ in terms of FU
$\rho_{p\rightarrow \mathrm{f}}$	scaling factor for elementary flow $\mathrm{LCI}^{\mathrm{up}}_{p,e}$ in terms of FU
$\boldsymbol{\varphi}$	vector of uncertain background flows
$\boldsymbol{\omega}$	vector of uncertain foreground parameters
$\sigma^{\mathrm{EF}}_{p,e}$	standard deviation in log-normal distribution of elementary flow $\mathrm{EF}_{p,e}$
$\hat{\sigma}_{\mathrm{EI}_z}$	sample standard-deviation of environmental impact EI_z

B. Property Estimation and Process Flowsheeting

This section reviews the properties used for the pseudo components in Aspen-HYSYS (version 9) for process simulation and how they are estimated.

- For estimating the enthalpies of formation, the molecular structure of the cation and anion are first drawn and optimized in the molecular modelling and graphics software ArgusLab. The structure is then processed in the open-source software MOPAC, a quantum chemistry tool for calculating the charge density profiles and enthalpies of formation.

- The heat of formation of ionic liquids is obtained from the Born-Haber cycle, as shown in Equation (S1) below.[2]

$$\Delta H^\circ_{\mathrm{f_{IL}}} = \Delta H^\circ_{\mathrm{f_{cation}}} + \Delta H^\circ_{\mathrm{f_{anion}}} - \Delta H_{\mathrm{L}} \tag{S1}$$

  The lattice energy ΔH_{L} is calculated from Equation (S2).[5] R is the ideal gas constant. n_{m} and n_{x} are parameters that depend on the nature of the cation and anion, respectively: they are equal to 3 for monoatomic ions, 5 for linear polyatomic ions, and 6 for non-linear polyatomic ions. p and q are the oxidation states of the cation and anion, respectively. The potential energy U_{pot} is calculated from Equation (S3), where ρ_{m} and M_{m} are is the density and the molecular weight of the ionic liquid, and γ and δ are coefficients that depend on the stoichiometry of the ionic liquid.

$$\Delta H_{\mathrm{L}} = U_{\mathrm{pot}} + \left[p\left(\frac{n_{\mathrm{m}}}{2} - 2\right) + q\left(\frac{n_{\mathrm{x}}}{2} - 2\right)\right] RT \tag{S2}$$

$$U_{\mathrm{pot}} = \gamma \left(\frac{\rho_{\mathrm{m}}}{M_{\mathrm{m}}}\right)^{1/3} + \delta \tag{S3}$$

Table S1: 1-methylimidazole properties

**Property**	**Value**	**Units**
MW	82	g mol^{-1}
BP	198[1]	°C
Density	1030[1]	kg m^{-3}
ΔH_{f}	125700[11]	kJ kmol^{-1}
T_{c}	490.90	°C
P_{c}	6086	kPa
V_{c}	0.26	m^3 kmol^{-1}
Acentricity	0.35	–

Table S2: [BMIM][BF_4] properties

**Property**	**Value**	**Units**
MW	226	g mol^{-1}
BP	222.05[10]	°C
Density	1208[10]	kg m^{-3}
ΔH_f	-650300	kJ $kmol^{-1}$
T_c	370.10[10]	°C
P_c	2038[10]	kPa
V_c	0.66[10]	m^3 $kmol^{-1}$
Acentricity	0.89[10]	–

Table S3: [BMIM][PF_6] properties

**Property**	**Value**	**Units**
MW	284	g mol^{-1}
BP	281[10]	°C
Density	1346[10]	kg m^{-3}
ΔH_f	-1760000	kJ $kmol^{-1}$
T_c	446[10]	°C
P_c	1728[10]	kPa
V_c	0.76[10]	m^3 $kmol^{-1}$
Acentricity	0.79[10]	–

Table S4: [BMIM][Cl] properties

**Property**	**Value**	**Units**
MW	175	g mol^{-1}
BP	285[10]	°C
Density	1080[10]	kg m^{-3}
ΔH_f	-237000	kJ kmol^{-1}
T_c	516[10]	°C
P_c	2785[10]	kPa
V_c	0.57[10]	m^3 kmol^{-1}
Acentricity	0.49[10]	–

Table S5: Sodium tetrafluoroborate properties

**Property**	**Value**	**Units**
MW	110	g mol^{-1}
BP	500	°C
Density	2470	kg/m^3
ΔH_f	-1850000	kJ kmol^{-1}
T_c	782	°C
P_c	2260	kPa
V_c	0.82	m^3 kmol^{-1}
Acentricity	0.59	–

Table S6: Lithium hexafluorophosphate properties

**Property**	**Value**	**Units**
MW	152	g mol^{-1}
BP	500	°C
Density	1500	kg/m^3
ΔH_f	-2300000[3]	kJ kmol^{-1}
T_c	782	°C
P_c	2258	kPa
V_c	0.80	m^3 kmol^{-1}
Acentricity	0.59	–

Table S7: Lithium chloride properties

**Property**	**Value**	**Units**
MW	42.4	g mol^{-1}
BP	1382	°C
Density	2070	kg/m^3
ΔH_f	-408300	kJ kmol^{-1}
T_c	1442	°C
P_c	1015	kPa
V_c	3.04	m^3 kmol^{-1}
Acentricity	2.52	–

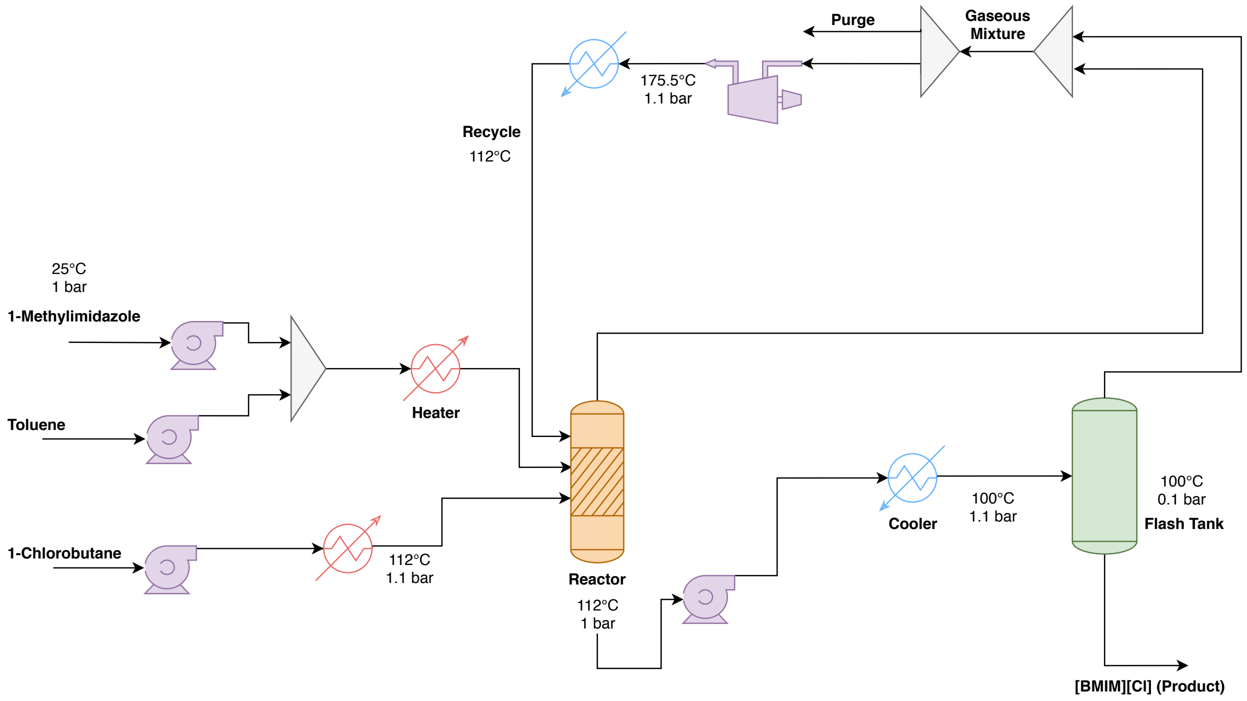

Figure S1: [BMIM][Cl] process flow diagram

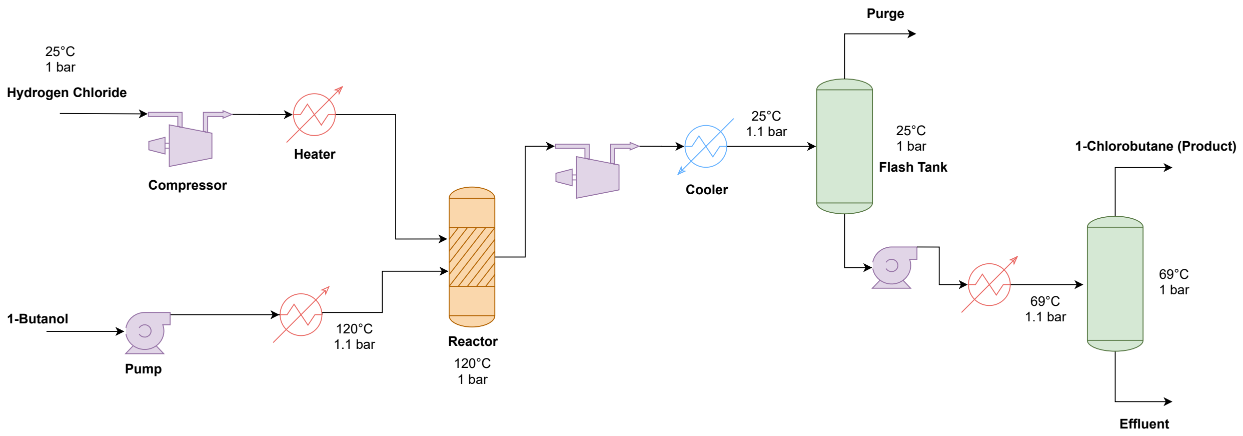

Figure S2: 1-Chlorobutane process flow diagram

C. Environmental Assessment Data and Methodology

This section gathers all data related to LCA including the proxy data, processes and flows used for the LCI phase in addition to the midpoint results from the characterization phase.

Table S8: Proxy data used in LCI

Data Category	Proxy data	Proxy method
Air emissions	Raw materials	0.2% by mass of inflows are assumed to be vaporized or leaked
	Cooling water	4% by volume of total cooling water are assumed to be vaporized or leaked
	CO_2	90% by mass of carbon in waste stream is assumed to be completely burned in waste treatment to produce CO_2 as per the following complete combustion equation: $C_\alpha H_\beta O_\gamma + (\alpha + \frac{\beta}{4} - \frac{\gamma}{2})\, O_2 \longrightarrow \alpha CO_2 + \frac{\beta}{2}\, H_2O$
Water emissions	COD	The chemical oxygen demand (COD) or total oxygen consumed is assumed to be equivalent to the amount of oxygen needed to react with the amount of carbon remaining in the waste stream after treatment which is assumed to be 10% of total carbon
	BOD	For worst case scenario, the biological oxygen demand (BOD) which is the oxygen consumed due to biological aerobic digestion by organisms is assumed to be equivalent to the amount of COD
	TOC	The total organic carbon (TOC) which is the total amount of carbon is assumed to be equivalent to 10% of the total carbon in the waste stream which is the amount of carbon remaining after treatment
	DOC	For waste case scenario, dissolved organic carbon (DOC) is assumed to be equivalent to TOC

Table S9: [BMIM][BF_4] inventory

**Group**	**Inventory**	**Flow** (per-kg product)	**STDEV**
Inputs from nature	Water, cooling, unspecified natural origin, RER	0.12 m^3	1.0502
	Water, river, RER	0.06 m^3	1.0502
	Water, well, in ground, RER	0.06 m^3	1.0502
Inputs from technosphere (materials)	1-Butyl-3-methylimidazolium chloride	1.16 kg	———
	Chemical factory, organics {GLO} | market for | Cut-off	4.00×10^{-10} p	2.9905
	Heat, district or industrial, natural gas {RER} | market group for | Cut-off	5.31 MJ	1.0502
	Electricity, medium voltage {RER} | market group for | Cut-off	2.11×10^{-4} kWh	1.0502
	Heat, from steam, in chemical industry {RER} | market for heat, from steam, in chemical industry | Cut-off	0.59 MJ	1.0502
	Sodium tetrafluoroborate {GLO} | market for | Cut-off	1.38 kg	1.3269
	Tap water {RER} | market group for | Cut-off	0.12 kg	1.3269
Emissions to air	Carbon dioxide, fossil	0.70 kg	1.0502
	Sodium tetrafluoroborate	5.02×10^{-3} kg	1.0502
	Water/m^3	4.78×10^{-3} m^3	1.0502
Emissions to water	BOD5, Biological Oxygen Demand	0.06 kg	1.4918
	COD, Chemical Oxygen Demand	0.06 kg	1.4918
	DOC, Dissolved Organic Carbon	0.02 kg	1.4918
	TOC, Total Organic Carbon	0.02 kg	1.4918
	Water, RER	0.12 m^3	1.0502
	Sodium chloride	0.27 kg	1.0502
	Imidazole	3.99×10^{-2} kg	1.0502
Outputs to technosphere	Wastewater, average {Europe without Switzerland} | market for wastewater, average | Cut-off, U	1.66×10^{-3} m^3	1.0502

Table S10: [BMIM][PF_6] inventory

Group	Inventory	Flow (per-kg product)	STDEV
Inputs from nature	Water, cooling, unspecified natural origin, RER	0.07 m^3	1.0502
	Water, river, RER	0.03 m^3	1.0502
	Water, well, in ground, RER	0.03 m^3	1.0502
Inputs from technosphere (materials)	1-Butyl-3-methylimidazolium chloride	0.89 kg	———
	Chemical factory, organics {GLO} | market for | Cut-off	4.00×10^{-10} p	2.9905
	Heat, district or industrial, natural gas {RER} | market group for | Cut-off	2.17 MJ	1.0502
	Electricity, medium voltage {RER} | market group for | Cut-off	1.32×10^{-4} kWh	1.0502
	Heat, from steam, in chemical industry {RER} | market for heat, from steam, in chemical industry | Cut-off	0.24 MJ	1.0502
	Lithium hexafluorophosphate {GLO} | market for | Cut-off	1.06 kg	1.3269
	Tap water {RER} | market group for | Cut-off	0.09 kg	1.3269
Emissions to air	Carbon dioxide, fossil	0.49 kg	1.0502
	Lithium hexafluorophosphate	2.11×10^{-3} kg	1.0502
	Water/m^3	2.86×10^{-3} m^3	1.0502
Emissions to water	BOD5, Biological Oxygen Demand	0.04 kg	1.4918
	COD, Chemical Oxygen Demand	0.04 kg	1.4918
	DOC, Dissolved Organic Carbon	0.01 kg	1.4918
	TOC, Total Organic Carbon	0.01 kg	1.4918
	Water, RER	0.12 m^3	1.0502
	Lithium	0.03 m^3	1.0502
	Chloride	0.13 kg	1.0502
	Imidazole	3.00×10^{-2} kg	1.0502
Outputs to technosphere	Wastewater, average {Europe without Switzerland} | market for wastewater, average | Cut-off, U	1.01×10^{-3} m^3	1.0502

Table S11: [BMIM][Cl] inventory

**Group**	**Inventory**	**Flow** (per-kg product)	**STDEV**
Inputs from nature	Water, cooling, unspecified natural origin, RER	5.92×10^{-3} m^3	1.0502
	Water, river, RER	2.96×10^{-3} m^3	0.03502
	Water, well, in ground, RER	2.96×10^{-3} m^3	0.03502
Inputs from technosphere (materials)	1-Methylimidazole	0.47 kg	1.3269
	Chemical factory, organics {GLO} | market for | Cut-off	4.00×10^{-10} p	2.9905
	Heat, district or industrial, natural gas {RER} | market group for | Cut-off	0.33 MJ	1.0502
	Electricity, medium voltage {RER} | market group for | Cut-off	1.79×10^{-4} kWh	1.0502
	Heat, from steam, in chemical industry {RER} | market for heat, from steam, in chemical industry | Cut-off	3.70×10^{-2} MJ	1.0502
	1-Chlorobutane	0.85 kg	1.3269
	Toluene, liquid {GLO} | market for | Cut-off, U	0.20 kg	1.3269
Emissions to air	Carbon dioxide, fossil	0.19 kg	1.0502
	Imidazole	0.03 kg	1.0502
	Chloride	7.70×10^{-3} kg	1.0502
	1,4-Dichloromethane	0.41 kg	1.0502
	Water/m^3	2.37×10^{-6} m^3	1.0502
	Toluene	0.39 kg	1.0502
Emissions to water	Water, RER	5.92×10^{-2} m^3	1.0502
	BOD5, Biological Oxygen Demand	1.57×10^{-2} kg	1.4918
	COD, Chemical Oxygen Demand	1.57×10^{-2} kg	1.4918
	DOC, Dissolved Organic Carbon	5.87×10^{-3} kg	1.4918
	TOC, Total Organic Carbon	5.87×10^{-3} kg	1.4918
	1,4-Dichloromethane	4.05×10^{-3} kg	1.0502
	Toluene	3.91×10^{-3} kg	1.0502
	Chloride	7.69×10^{-5} kg	1.0502
	Imidazole	3.02×10^{-4} kg	1.0502
Outputs to technosphere	Wastewater, average {Europe without Switzerland} | market for wastewater, average | Cut-off, U	8.34×10^{-5} m^3	1.0502

Table S12: 1-Chlorobutane inventory

Group	Inventory	Flow (per-kg product)	STDEV
Inputs from nature	Water, cooling, unspecified natural origin, RER	4.68×10^{-2} m^3	1.0502
	Water, river, RER	2.34×10^{-2} m^3	0.03502
	Water, well, in ground, RER	2.34×10^{-2} m^3	0.03502
Inputs from technosphere (materials)	Hydrochloric acid, without water, in 30% solution state {RER} | hydrochloric acid production, from the reaction of hydrogen with chlorine | Cut-off, U	0.94 kg	1.3269
	Chemical factory, organics {GLO} | market for | Cut-off	4.00×10^{-10} p	2.9905
	Heat, district or industrial, natural gas {RER} | market group for | Cut-off	0.31 MJ	1.0502
	Electricity, medium voltage {RER} | market group for | Cut-off	2.48×10^{-2} kWh	1.0502
	Heat, from steam, in chemical industry {RER} | market for heat, from steam, in chemical industry | Cut-off	3.50×10^{-2} MJ	1.0502
	1-butanol {GLO} | market for | Cut-off, U	1.52 kg	1.3269
Emissions to air	Carbon dioxide, fossil	1.21 kg	1.0502
	Hydrogen chloride	0.31 kg	1.0502
	1-Butanol	5.23×10^{-3} kg	1.0502
	1,4-Dichlorbutane	0.11 kg	1.0502
	Water	7.51×10^{-5} m^3	1.0502
Emissions to water	Water, RER	0.05 m^3	1.0502
	BOD5, Biological Oxygen Demand	9.77×10^{-2} kg	1.4918
	COD, Chemical Oxygen Demand	9.77×10^{-2} kg	1.4918
	DOC, Dissolved Organic Carbon	3.66×10^{-2} kg	1.4918
	TOC, Total Organic Carbon	3.66×10^{-2} kg	1.4918
	1,4-Dichlorobutane	4.07×10^{-2} kg	1.0502
	1-Butanol	2.37×10^{-2} kg	1.0502
Outputs to technosphere	Wastewater, average {Europe without Switzerland} | market for wastewater, average | Cut-off, U	8.40×10^{-4} m^3	1.0502

Table S13: LCA ReCiPe midpoint results, for 1 kg of ionic liquid

**Impact indicator**	**Unit**	**[BMIM][BF$_4$]**	**[BMIM][PF$_6$]**
Global warming	kg CO_2 eq	27.3	32.5
Stratospheric ozone depletion	kg CFC11 eq	1.04×10^{-5}	1.17×10^{-5}
Ionizing radiation	kBq Co-60 eq	2.18	2.29
Ozone formation, Human health	kg NO_x eq	0.13	0.12
Fine particulate matter formation	kg PM2.5 eq	0.05	0.07
Ozone formation, Terrestrial ecosystems	kg NO_x eq	0.17	0.16
Terrestrial acidification	kg SO_2 eq	0.11	0.16
Freshwater eutrophication	kg P eq	1.05×10^{-2}	1.30×10^{-2}
Marine eutrophication	kg N eq	1.17×10^{-2}	1.14×10^{-2}
Terrestrial ecotoxicity	kg 1,4-DCB eq	74	131
Freshwater ecotoxicity	kg 1,4-DCB eq	0.79	1.16
Marine ecotoxicity	kg 1,4-DCB eq	1.12	1.66
Human carcinogenic toxicity	kg 1,4-DCB eq	0.95	1.21
Human non-carcinogenic toxicity	kg 1,4-DCB eq	24.3	38.7
Land use	m^2a crop eq	0.44	0.82
Mineral resource scarcity	kg Cu eq	0.07	0.64
Fossil resource scarcity	kg oil eq	9.40	9.76
Water consumption	m^3	1.02	0.98

Table S14: LCA ReCiPe endpoint results, for 1 kg of ionic liquid

**Impact indicator**	**Unit**	**[BMIM][BF$_4$]**	**[BMIM][PF$_6$]**
Human health	DALY	6.81×10^{-5}	8.64×10^{-5}
Ecosystem quality	species×yr	1.49×10^{-7}	1.78×10^{-7}
Resources availability	US$_{2020}$	2.91	3.24

D. Uncertainty Analysis Data and Comparison

This section reports the uncertain parameters and their corresponding errors for simulating the production of ionic liquids and their precursors. It also compares the probability for [BMIM][BF_4] production to have higher impacts than [BMIM][PF_6] production under sampled uncertainty scenarios.

Table S15: Uncertain model parameters, uncertainty sources and ranges in flowsheet simulation of [BMIM][PF_6] production. Each uncertain parameter is assumed to follow a triangular distribution.

Source	Parameter	Range	Units
Process	$\Delta P_{\mathrm{R}}{}^{a}$	10±50%	kPa
	$\Delta P_{\mathrm{W}}{}^{a}$	10±50%	kPa
	$T_{\mathrm{VF}}{}^{b}$	95±20%	°C
	$P_{\mathrm{VF}}{}^{a}$	10±50%	kPa
	PUR^{a}	0.1±50%	–
Molecular	$\rho_{[\mathrm{BMIM}][\mathrm{PF_6}]}{}^{c}$	1346±19%	$\mathrm{kg\,m^{-3}}$
	$\rho_{[\mathrm{BMIM}][\mathrm{Cl}]}{}^{c}$	1080±19%	$\mathrm{kg\,m^{-3}}$
	$\Delta H_{\mathrm{f}\,[\mathrm{BMIM}][\mathrm{PF_6}]}{}^{d}$	$-1.76\pm0.16 \times 10^{6}$	$\mathrm{kJ\,kmol^{-1}}$
	$\Delta H_{\mathrm{f}\,[\mathrm{BMIM}][\mathrm{Cl}]}{}^{d}$	$-2.37\pm1.59 \times 10^{5}$	$\mathrm{kJ\,kmol^{-1}}$

[a] Estimate based on heuristics.
[b] Mean value based on an optimized base case.
[c] Estimate based on the group contribution methods developed by Valderrama and Rojas[10] with maximum standard deviation of 19 %.
[d] Estimate based on the lattice energy and computational chemistry methods proposed by Gao et al.[2] with maximum deviation of $-159\,\mathrm{kJ\,mol^{-1}}$.

Table S16: Uncertain model parameters, uncertainty sources and ranges in flowsheet simulation of [BMIM][Cl] production. Each uncertain parameter is assumed to follow a triangular distribution.

**Source**	**Parameter**	**Range**	**Units**
Process	ΔP_{R}[a]	10±50%	kPa
	T_{VF}[b]	100±20%	°C
	P_{VF}[a]	10±50%	kPa
Molecular	$\rho_{\mathrm{[BMIM][Cl]}}$[c]	1080±19%	$\mathrm{kg\,m^{-3}}$
	$\Delta H_{\mathrm{f\,[BMIM][Cl]}}$[d]	-2.37±1.59×10^5	$\mathrm{kJ\,kmol^{-1}}$

[a] Estimate based on heuristics.
[b] Mean value based on an optimized base case.
[c] Estimate based on the group contribution methods developed by Valderrama and Rojas[10] with maximum standard deviation of 19 %.
[d] Estimate based on the lattice energy and computational chemistry methods proposed by Gao et al.[2] with maximum deviation of $-159\,\mathrm{kJ\,mol^{-1}}$.

Table S17: Uncertain model parameters, uncertainty sources and ranges in flowsheet simulation of 1-chlorobutane production. Each uncertain parameter is assumed to follow a triangular distribution.

**Source**	**Parameter**	**Range**	**Units**
Process	ΔP_{R}[a]	10±50%	kPa
	ΔP_{F1}[a]	10±50%	kPa
	ΔP_{F2}[a]	10±50%	kPa
	F_{HCl}[b]	37.5±10%	$\mathrm{kmol\,h^{-1}}$
	$F_{\text{1-butanol}}$[b]	30±50%	$\mathrm{kmol\,h^{-1}}$
	X_{R}[c]	0.8±10%	–

[a] Estimate based on heuristics.
[b] Mean value based on an optimized base case.
[c] Estimate based on yield values reported in the literature.

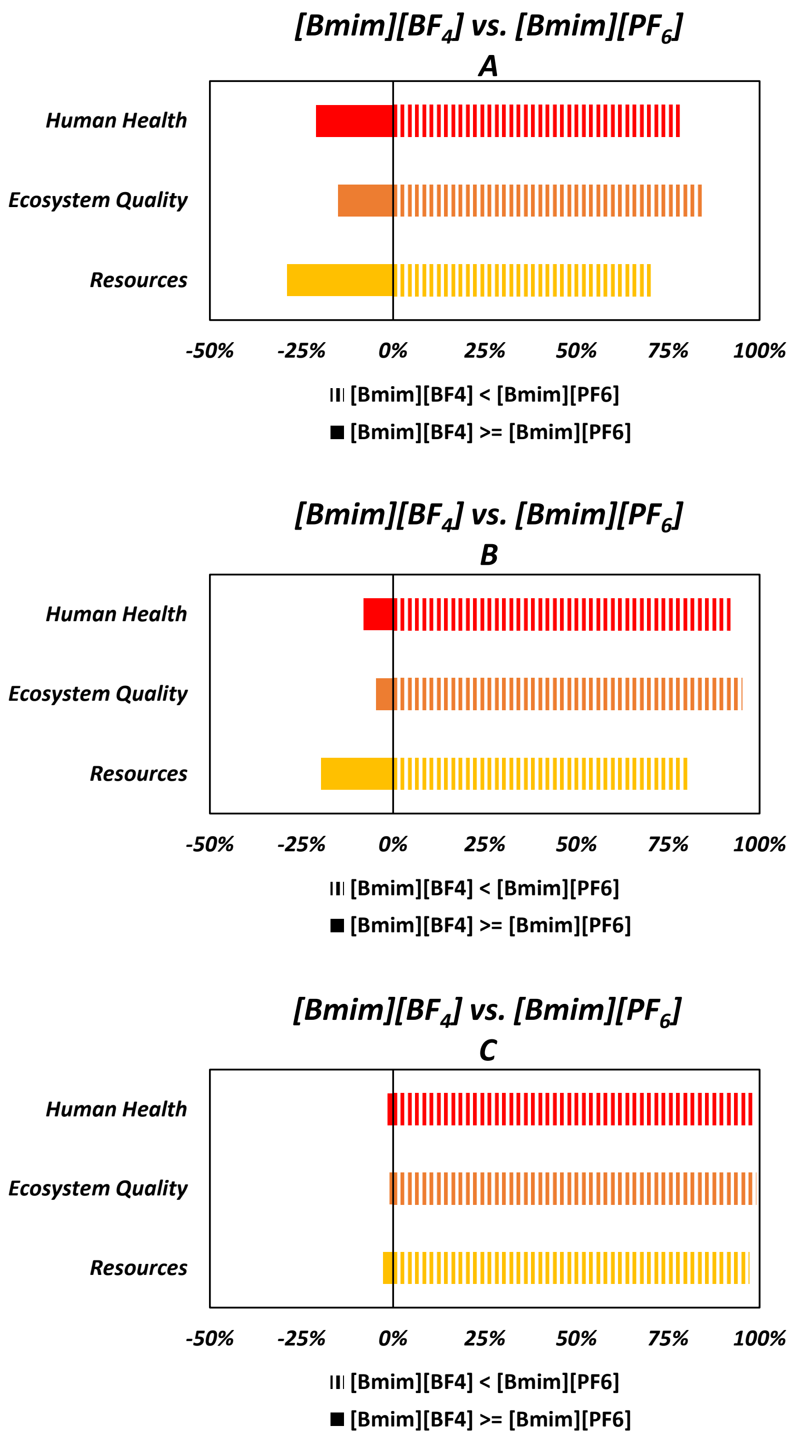

Figure S3: Overall scenario-based comparison between the end-point impacts of [BMIM][BF_4] and [BMIM][PF_6] under combined foreground/background uncertainty (A), background uncertainty only (B), and foreground uncertainty only (C).

E. Uncertainty Quantification Methodology and Results

This section provides further details about the computational methodology used for uncertainty quantification and apportionment using the RS-HDMR method of GSA. It also presents a comparison with the parameter rankings obtained using one-at-a-time sensitivity analysis.

- The uncertainty propagation was coordinated in Matlab. An XML file was created from the database ecoinvent (version 3.5) and imported to Matlab. This file was processed in order to retrieve the background processes and their connectivity as well as the corresponding background uncertainties. All these background uncertainties together with the foreground process uncertainties (Tables 1, S15, S16 & S17) were jointly sampled using quasi-random Sobol sequences.[9] For each sample, the foreground inventory flows were first computed using Aspen-HYSYS (version 9, interfaced with Matlab). These flows were then propagated through the background processes. The resulting elementary and intermediate flows were combined into total life-cycle inventories $\mathrm{LCI}_e^{\mathrm{tot}}$ for each elementary flow $e \in E$ (Equation 2), further combined into environmental impacts EI_z for each category $z \in Z$ (Equation 1) using the characterization factors $\mathrm{CF}_{e,z}$ in the ReCiPe 2016 methodology[4]. The elementary and intermediate flows were also combined to compute the lumped background uncertainties $\mathrm{BEI}_{p,z}^{\mathrm{up}}$ for relevant processes p in the immediate background (Equation 7).

- The variance-based global sensitivity analysis (GSA) was conducted using SobolGSA (version 3.1.1)[6,7] under Matlab. A separate GSA was conducted for each end-point impact z, using the sampled environmental impacts EI_z as output values and the corresponding foreground uncertainty $\boldsymbol{\omega}$ and lumped background uncertainties $\mathrm{BEI}_{p,z}^{\mathrm{up}}(\boldsymbol{\varphi})$ as input scenarios. In doing so, notice that any cross-correlations between the uncertain inputs are indeed captured—as opposed to treating $\boldsymbol{\omega}$ and $\mathrm{BEI}_{p,z}^{\mathrm{up}}(\boldsymbol{\varphi})$ as independent uncertainties. Denoting the uncertainties as $u_1, \ldots, u_d$ for simplicity, The ANOVA-based decomposition of EI_z is given by:

$$\mathrm{EI}_z = \mathrm{EI}_{z,0} + \sum_{i=1}^{n} \mathrm{EI}_{z,i}(u_i) + \sum_{1\le i<j\le n} \mathrm{EI}_{z,i,j}(u_i, u_j) + \cdots + \mathrm{EI}_{z,1,2,\ldots,d}(u_1, u_2, \ldots, u_d)$$

  The zeroth-order term $\mathrm{EI}_{z,0}$, first-order terms $\mathrm{EI}_{z,i}$ and second-order terms $\mathrm{EI}_{z,i,j}$ in this decomposition were computed using the RS-HDMR method through SobolGSA. This method exploits the fact that for many practical models only low-order interactions between inputs carry a significant impact on the output.[8] The first- and second-order terms are approximated using truncated series expansion:

$$\mathrm{EI}_{z,i}(u_i) \approx \sum_{k=1}^{K} \alpha_k^i \phi_k(\boldsymbol{\gamma_i}) \tag{S4}$$

$$\mathrm{EI}_{z,i,j}(u_i, u_j) \approx \sum_{m=1}^{M} \sum_{n=1}^{N} \beta_{mn}^{ij} \phi_m(u_i)\phi_n(u_j) \tag{S5}$$

where the choice of the orthogonal polynomial basis ϕ_k depends on the probability distributions of the inputs—see, e.g., Wang et al.[12] for the case of triangular distributions. The polynomial orders K, M and N are determined automatically by SobolGSA for maximal accuracy. Moreover, the coefficients α_k^i and β_{mn}^{ij} can be estimated via regression and directly feed into the following formulas to estimate the partial variances of the first- and second-order effects, respectively:

$$\mathrm{Var}[\mathrm{EI}_{z,i}(u_i)] \approx \sum_{k=1}^{K} (\alpha_k^i)^2 \tag{S6}$$

$$\mathrm{Var}[\mathrm{EI}_{z,i,j}(u_i, u_j)] \approx \sum_{m=1}^{M} \sum_{n=1}^{N} (\beta_{mn}^{ij})^2 \tag{S7}$$

In turn, the first- and second-order Sobol indices can be computed as:

$$\mathrm{SO}_{z,i} = \frac{\mathrm{Var}[\mathrm{EI}_{z,i}(u_i)]}{\mathrm{Var}[\mathrm{EI}_z]} \tag{S8}$$

$$\mathrm{SO}_{z,i,j} = \frac{\mathrm{Var}[\mathrm{EI}_{z,i,j}(u_i, u_j)]}{\mathrm{Var}[\mathrm{EI}_z]} \tag{S9}$$

where the total variance $\mathrm{Var}[\mathrm{EI}_z]$ can be estimated directly from the output samples. Lastly, the total-order Sobol index for a given input u_i as:

$$\mathrm{SO}_{z,i,\mathrm{tot}} = \mathrm{SO}_{z,i} + \mathrm{SO}_{z,i,1} + \cdots + \mathrm{SO}_{z,i,d} \tag{S10}$$

In practice, $\mathrm{SO}_{z,i,\mathrm{tot}}$ quantifies how much of the variance in environmental impacts EI_z may be attributed to the uncertainty in input u_i, either separately or in association with other uncertain inputs. When the difference between the first- and total-order Sobol index is small, $\mathrm{SO}_{z,i,\mathrm{tot}} \approx \mathrm{SO}_{z,i}$, the effect of the input u_i on the output variance is essentially separable from, and therefore additive to, the effect of the other inputs.

- By contrast to GSA, the one-at-a-time sensitivity analysis (OTSA) was performed by sampling each uncertain foreground parameter separately, while keeping the rest of the (foreground and background) parameters at their nominal values. Moreover, the resulting output variance for each input u_i was normalized by the total variance $\mathrm{Var}[\mathrm{EI}_z]$ from the joint uncertainty propagation (see above), so the resulting index could be directly compared with the total-order Sobol index for the same input.

Comparisons between the total-order Sobol indices and the OTSA indices for each foreground parameter and each end-point impact in the production of [BMIM][BF_4] and [BMIM][PF_6] are presented in Tables S18 and S19, respectively. These reveal massive differences in relative sensitivity of the background parameters, confirming the need to account for interaction effects and for a global analysis in general.

Table S18: Comparison between the total-order Sobol indices and the OTSA indices for each foreground parameter (rows) and each end-point impact (columns) in the production of [BMIM][BF_4].

**Parameter**	**Human Health**		**Ecosystem Quality**		**Resources**	
	**OAT**	**GSA**	**OAT**	**GSA**	**OAT**	**GSA**
ΔP_{R}	0.03	0.06	0.03	0.06	0.03	0.06
ΔP_{W}	0.03	0.05	0.03	0.06	0.03	0.05
T_{VF}	0.19	0.82	0.18	0.71	0.18	0.75
P_{VF}	0.08	0.61	0.11	0.59	0.10	0.61
PUR	0.10	0.20	0.11	0.20	0.11	0.20
$\rho_{\mathrm{[BMIM][BF_4]}}$	0.00	0.00	0.00	0.00	0.00	0.00
$\rho_{\mathrm{[BMIM][Cl]}}$	0.00	0.00	0.00	0.00	0.00	0.00
$\Delta H_{\mathrm{f[BMIM][BF_4]}}$	0.04	0.08	0.05	0.08	0.04	0.08
$\Delta H_{\mathrm{f[BMIM][Cl]}}$	0.05	0.09	0.05	0.08	0.05	0.08

Table S19: Comparison between the total-order Sobol indices and the OTSA indices for each foreground uncertainty (rows) and each end-point impact (columns) in the production of [BMIM][PF_6].

**Parameter**	**Human Health**		**Ecosystem Quality**		**Resources**	
	**OAT**	**GSA**	**OAT**	**GSA**	**OAT**	**GSA**
ΔP_{R}	0.03	0.05	0.03	0.05	0.03	0.05
ΔP_{W}	0.03	0.05	0.03	0.05	0.03	0.05
T_{VF}	0.20	0.75	0.19	0.69	0.19	0.70
P_{VF}	0.09	0.57	0.11	0.55	0.11	0.56
PUR	0.11	0.19	0.12	0.20	0.12	0.20
$\rho_{\mathrm{[BMIM][PF_6]}}$	0.00	0.00	0.00	0.00	0.00	0.00
$\rho_{\mathrm{[BMIM][Cl]}}$	0.00	0.00	0.00	0.00	0.00	0.00
$\Delta H_{\mathrm{f[BMIM][PF_6]}}$	0.05	0.09	0.05	0.09	0.05	0.09
$\Delta H_{\mathrm{f[BMIM][Cl]}}$	0.05	0.09	0.05	0.08	0.05	0.09

Table S20: Total-order Sobol indices for each immediate lumped background parameter (rows) and each end-point impact (columns) in the production of $NaBF_4$. The first- and total-order Sobol indices are identical since this subproblem is linear.

**Parameter**	**Human Health**	**Ecosystem Quality**	**Resources**
$\mathrm{BEI}_z^{\mathrm{BF_3}}$	0.60	0.58	0.60
$\mathrm{BEI}_z^{\mathrm{mat}}$	0.03	0.02	0.01
$\mathrm{BEI}_z^{\mathrm{Et_2O}}$	0.03	0.04	0.08
$\mathrm{BEI}_z^{\mathrm{el}}$	0.05	0.05	0.03
$\mathrm{BEI}_z^{\mathrm{th}}$	0.00	0.01	0.03
$\mathrm{BEI}_z^{\mathrm{NaF}}$	0.29	0.30	0.25

Table S21: Total-order Sobol indices for each immediate lumped background parameter (rows) and each end-point impact (columns) in the production of $LiPF_6$. The first- and total-order Sobol indices are identical since this subproblem is linear.

**Parameter**	**Human Health**	**Ecosystem Quality**	**Resources**
$\mathrm{BEI}_z^{\mathrm{HF}}$	0.45	0.43	0.44
$\mathrm{BEI}_z^{\mathrm{mat}}$	0.03	0.02	0.01
$\mathrm{BEI}_z^{\mathrm{Cl_5P}}$	0.24	0.28	0.29
$\mathrm{BEI}_z^{\mathrm{el}}$	0.05	0.06	0.03
$\mathrm{BEI}_z^{\mathrm{th}}$	0.01	0.01	0.04
$\mathrm{BEI}_z^{\mathrm{LiF}}$	0.22	0.20	0.19

References

[1] 1-Methylimidazole M8878. URL `https://www.sigmaaldrich.com/catalog/product/sigma/m8878`.

[2] H. Gao, C. Ye, C. M. Piekarski, and J. M. Shreeve. Computational characterization of energetic salts. *The Journal of Physical Chemistry C*, 111(28):10718–10731, 2007. doi: 10.1021/jp070702b.

[3] K. S. Gavritchev, G. A. Sharpataya, A. A. Smagin, E. N. Malyi, and V. A. Matyukha. Calorimetric study of thermal decomposition of lithium hexafluorophosphate. *Journal of Thermal Analysis & Calorimetry*, 73(1):71–83, 2003. ISSN 1572-8943. doi: 10.1023/A:1025125306291.

[4] M. A. J. Huijbregts, Z. J. N. Steinmann, P. M. F. Elshout, G. Stam, F. Verones, M. Vieira, M. Zijp, A. Hollander, and R. van Zelm. ReCiPe2016: a harmonised life cycle impact assessment method at midpoint and endpoint level. *The International Journal of Life Cycle Assessment*, 22(2):138–147, 2017. doi: 10.1007/s11367-016-1246-y.

[5] H. D. B. Jenkins, D. Tudela, and L. Glasser. Lattice potential energy estimation for complex ionic salts from density measurements. *Inorganic Chemistry*, 41(9):2364–2367, 2002. doi: 10.1021/ic011216k.

[6] S. Kucherenko. SOBOLHDMR: A general-purpose modeling software. In K. M. Polizzi and C. Kontoravdi, editors, *Synthetic Biology*, pages 191–224, Totowa, NJ, 2013. Humana Press. doi: 10.1007/978-1-62703-625-2_16.

[7] S. Kucherenko and O. Zaccheus. SobolGSA Software. URL `http://www.imperial.ac.uk/a-z-research/process-systems-engineering/research/free-software/sobolgsa-software/`.

[8] H. Rabitz, Ö. F. Aliş, J. Shorter, and K. Shim. Efficient input-output model representations. *Computer Physics Communications*, 117(1):11–20, 1999. doi: 10.1016/S0010-4655(98)00152-0.

[9] I. M. Sobol. On the distribution of points in a cube and the approximate evaluation of integrals. *USSR Computational Mathematics & Mathematical Physics*, 7(4):86–112, 1967. doi: 10.1016/0041-5553(67)90144-9.

[10] J. O. Valderrama and R. E. Rojas. Critical properties of ionic liquids. Revisited. *Industrial & Engineering Chemistry Research*, 48(14):6890–6900, 2009. doi: 10.1021/ie900250g.

[11] S. P. Verevkin, D. H. Zaitsau, V. N. Emel'yanenko, Y. U. Paulechka, A. V. Blokhin, A. B. Bazyleva, and G. J. Kabo. Thermodynamics of ionic liquids precursors: 1-Methylimidazole. *Journal of Physical Chemistry B*, 115(15):4404–4411, 2011. doi: 10.1021/jp201752j.

[12] S. W. Wang, P. G. Georgopoulos, G. Li, and H. Rabitz. Random sampling-high dimensional model representation (RS-HDMR) with nonuniformly distributed variables: Application to an integrated multimedia/multipathway exposure and dose model for trichloroethylene. *The Journal of Physical Chemistry A*, 107(23):4707–4716, 2003. doi: 10.1021/jp022500f.
